# A simplified preparation method for single-nucleus RNA-sequencing using long-term frozen brain tumor tissues

**DOI:** 10.1038/s41598-025-97053-9

**Published:** 2025-04-14

**Authors:** Kati J. Ernst, Konstantin Okonechnikov, Josephine Bageritz, Ashwyn A. Perera, Jan-Philipp Mallm, Andrea Wittmann, Kendra K. Maaß, Svenja Leible, Michael Boutros, Stefan M. Pfister, Marc Zuckermann, David T. W. Jones

**Affiliations:** 1 Division of Pediatric Glioma Research, Hopp Children’S Cancer Center Heidelberg (Kitz), Heidelberg, Germany; 2 National Center for Tumor Diseases (NCT), NCT Heidelberg, a Partnership Between DKFZ and Heidelberg University Hospital, Heidelberg, Germany; 3 German Cancer Research Center (DKFZ), Heidelberg, Germany; 4 Division of Pediatric Neuro-Oncology, Hopp Children’S Cancer Center Heidelberg (Kitz), Heidelberg, Germany; 5 Division of Signaling and Functional Genomics, German Cancer Research Center (DKFZ), Heidelberg, Germany; 6 Heidelberg Medical Faculty, University of Heidelberg, Heidelberg, Germany; 7 Single-Cell Open Lab; German Cancer Research Center (DKFZ), Heidelberg, Germany; 8 Department of Pediatric Hematology and Oncology, University Hospital Heidelberg, Heidelberg, Germany

**Keywords:** Biological techniques, Cancer, Computational biology and bioinformatics

## Abstract

Single-cell RNA-sequencing has provided intriguing new insights into research areas such as developmental processes and tumor heterogeneity. Most approaches, however, rely on the availability of fresh surgical specimens, thereby dramatically reducing the ability to profile particularly rare tissue types. Here, we optimized a method to isolate intact nuclei from long-term frozen pediatric glioma tissues. We performed a technical comparison between different single-nucleus RNA-sequencing (snRNA-seq) systems and applied the established nucleus isolation method to analyze frozen primary glioma tissues. The results show that our fast, simple and low-cost nuclear isolation protocol provides intact nuclei, which can be used in both droplet- and plate-based single-cell sequencing platforms – allowing the identification of distinct tumor cell populations and infiltrating microglia. Additional optimization to include shorter RNA fragments in the 3’ sequencing library improved gene detection and cell type annotation. Taken together, the method dramatically increases the potential of studying rare tumor entities and is specifically tailored for using frozen brain tumor tissue.

## Introduction

Single-cell RNA-sequencing (scRNA-seq) is an increasingly popular method for investigating properties of heterogeneous tissues. Importantly, it allows the assessment of tumor heterogeneity and the identification of diverse tumor and non-tumor cell populations in a tumor mass, which cannot easily be addressed with conventional bulk tissue RNA-sequencing. Whole-cell extractions for traditional scRNA-seq, however, require viable fresh tissue, whereas often only frozen tumor biopsy samples are readily available.

The need for modern techniques that enable a thorough investigation and understanding of tumor biology is underlined by the clinical challenge presented by many tumor entities, such as childhood brain tumors. Central nervous system tumors (e.g. gliomas) are currently the leading cause of cancer-related morbidity and mortality in children in spite of all therapeutic efforts^1–4^. While there have been several studies looking at single-cell analysis of pediatric brain tumors from viable whole cells^5–18^application to frozen tissue (which would open up a wealth of possible samples) has been limited to date. We therefore sought to develop a robust method for profiling of single nuclei RNA-seq (snRNA-seq) extracted from long-term frozen samples of pediatric brain tumors. Previous reports have indicated the utility of frozen material for snRNA-seq^19–27^but there is still need for an enhanced nucleus isolation technique from frozen central nervous system tumors, since brain tissues are especially problematic as starting material due to their high neuron composition and thus increased sensitivity to enzymatic cell dissociation^28^.

Here, we present a nuclear isolation protocol specifically optimized for long-term frozen brain tumor tissues and an example workflow for identifying cell populations from snRNA-seq data. To study the usability of the nuclear preparations obtained with our protocol, we profiled the extracted nuclei using different snRNA-seq platforms: Chromium 10X Genomics^29^, Drop-seq^30^and Fluidigm C1 System^31^, in combination with several tumor types. The resulting data were further analyzed computationally, showing the applicability of the derived data for identification of cellular subpopulations; assignment to cell types; copy number analysis and evaluation of lineage hierarchies. In addition, we analyzed a short-fragment 10X cDNA library alongside the standard library to study its applicability in detecting shorter genes and transcripts.

## Results

### Isolation of intact nuclei with a good yield from long-term frozen pediatric glioma tissues

We first tested the density gradient centrifugation method originally developed by Spalding et. al. (2005)^32^and modified by Ernst et. al. (2014)^33^, and observed substantial cell debris and a low nuclear yield in the final preparation when using frozen human brain tumor tissues. In addition, we speculated that the extended processing time increased the risk of further RNA degradation during sample handling. To improve nucleus yield and quality, we modified the respective buffers, optimized the extent of homogenization through douncing, added two filtering steps after the cell lysis and adapted the ultracentrifugation step. Despite these measures, we found that the sample purity was still insufficient as assessed by visually inspecting debris fractions and morphology of nuclei (Supplementary Fig. S1a online). We therefore replaced the density gradient centrifugation with washing steps using lysis buffer without detergent, to avoid nuclear wall permeabilization. Although this observably improved purity, the yield remained low. To overcome this, we tested several consumable plastics, coating buffers and buffer volumes and finally managed to increase the yield of nuclei (Supplementary Fig. S1e online; see Materials & Methods). The buffer volumes were adjusted based on the amount of starting material (here we present volumes suitable for about 20–50 mg of frozen glioma tissue). During the processing it was observed that soft tumors with a fairly uniform mass of cancer cells resulted in a higher yield than tumors that were interspersed by neurons and harder to dissociate, and that brainstem tissues resulted generally in a lower yield of nuclei than tissues from cortical areas, although this was not rigorously quantified. An overview comparison of the original and optimized protocol is shown in Supplementary Table S1 online.

As a comparison, we also tested three commercial nuclear isolation methods: Nuclei EZ Prep (Sigma-Aldrich, NUC101 - 1 KT), Isolation of Nuclei for Single-Cell RNA Sequencing^34^and OptiPrepTM^35^. The EZ prep resulted in a high yield, but also in a very high number of debris in the supernatant (Supplementary Fig. S1b online), while the 10X Genomics protocol gave a clear supernatant but very low yield of nuclei (Supplementary Fig. S1c online). When replacing the washing step of our protocol with OptiPrep™ density gradient, we obtained similar results as when using our original sucrose cushion density gradient, but with a slightly lower yield (Supplementary Fig. S1 d online). Our optimized protocol therefore provides a good balance between purity and yield, in addition to the benefits of its simplicity and cost-effectiveness.

**Fig. 1:**
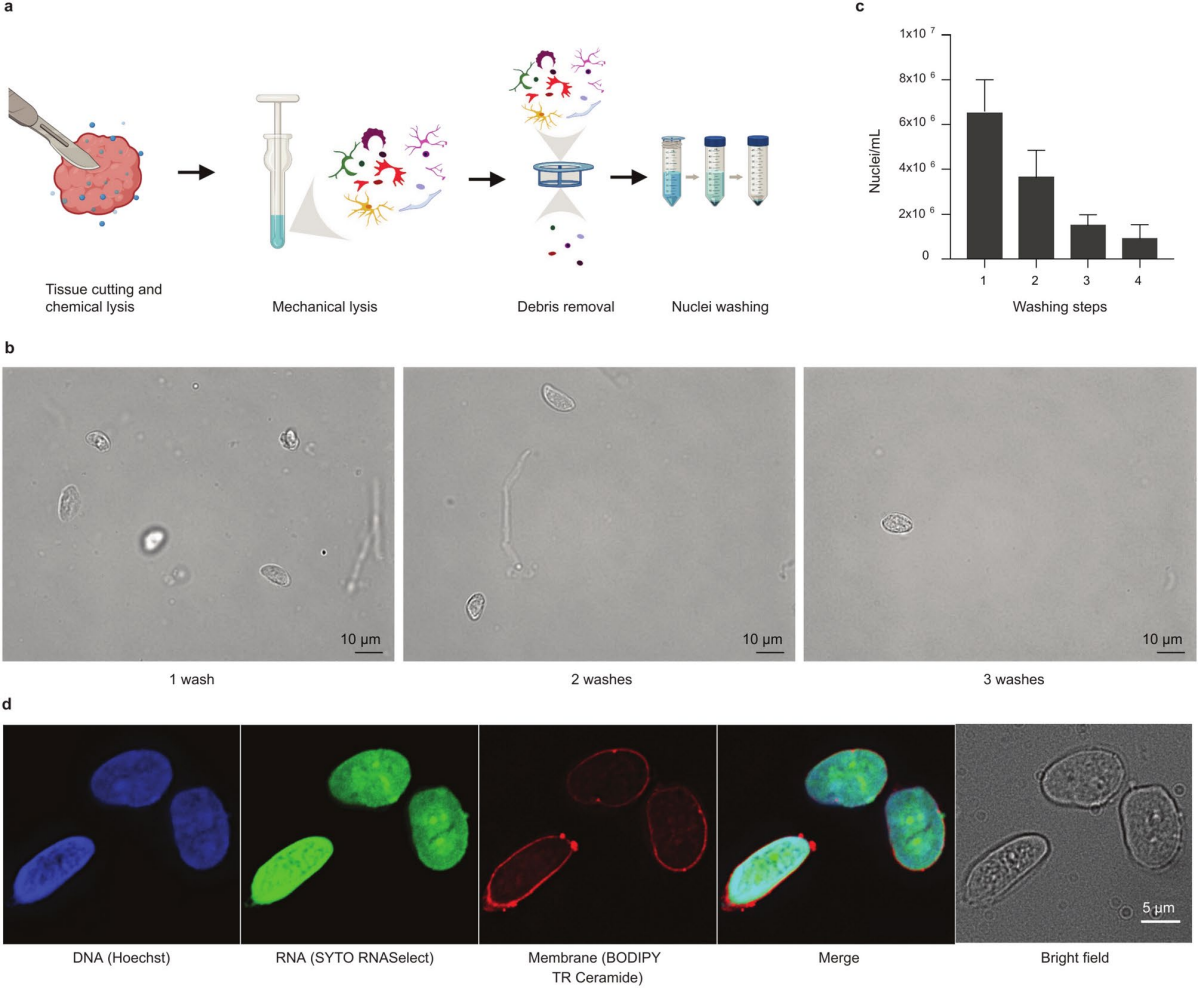
Isolation of intact nuclei from long-term frozen pediatric glioma tissue. (**a**) Schematic figure showing sample preparation steps. (**b**) Representative images showing the effect of an increasing number of washing steps on nuclei yield/integrity and number of debris (scale bar 10 μm). (**c**) Nuclei yield decreases with increasing number of washes. Data are represented as mean with SEM. (**d**) Staining for nuclear membrane, DNA and RNA reveals intact nuclei, with no leakage of nucleic acids (scale bars 5 μm). Nuclei originate from a pediatric pilocytic astrocytoma tissue frozen for seven years prior to nuclear extraction. Created with BioRender.com, GraphPad Prism 5 and Affinity Designer 2.

Our final protocol is fast (less than 30 min in total), low-cost, and simple to carry out (a detailed operating protocol described in Supplementary Note 1 online). It consists of four steps: cutting the tissue in ice-cold lysis buffer with a scalpel, douncing the sample to open cell walls, filtering the cell debris and washing off the rest of the cell debris and free RNA (Fig. 1a). The sample can then be re-suspended in storage buffer and either applied directly for use with snRNA-seq platforms or frozen for a short period (maximally 2–3 days) at − 80 °C. Washing of the nuclei three times was found to be optimal for obtaining a debris-free supernatant (Fig. 1b), with further washing steps leading to damage of the nuclear walls. However, some nuclei are lost in each wash (Fig. 1c) and therefore two washes may be preferred if the amount of starting material is low. The protocol yields intact nuclei with undamaged nuclear walls in a debris-free supernatant (Fig. 1d). A further indication for the high quality of the prep is the very low proportions of mitochondrially-mapping reads in the resulting RNA data, with median proportions per cell typically under 1% (range 0.07–1.24%).

### Comparison of platforms for studying tumor heterogeneity using patient-derived xenografts

To investigate whether the nucleus material gives high-quality data when using different scRNA-seq platforms, we analyzed a pediatric glioblastoma patient-derived xenograft (PDX) sample (Supplementary Table S2 online) using Chromium 10X Genomics Single Cell Gene Expression v2.0 kit, Drop-seq (according to Bageritz et al. (2019)^36^) and Fluidigm C1 systems. Currently, PDXs are commonly used in preclinical studies^37^, and therefore it is important to also estimate the possibilities to computationally distinguish nuclei from the two different species. Proportions of covered reads aligned to exonic, intronic, intergenic and External RNA Controls Consortium (ERCC) reference types of three different snRNA-seq platforms were fairly similar, with the exception of ERCC spike-ins with Fluidigm C1 data (Supplementary Fig. S2a online). As expected, the highest number of nuclei was detected with 10X Genomics, with Drop-seq detecting only slightly less. The lower throughput design of the plate based Fluidigm C1 platform generally produces a reduced number of analyzed nuclei (Supplementary Table S3 online).

**Fig. 2:**
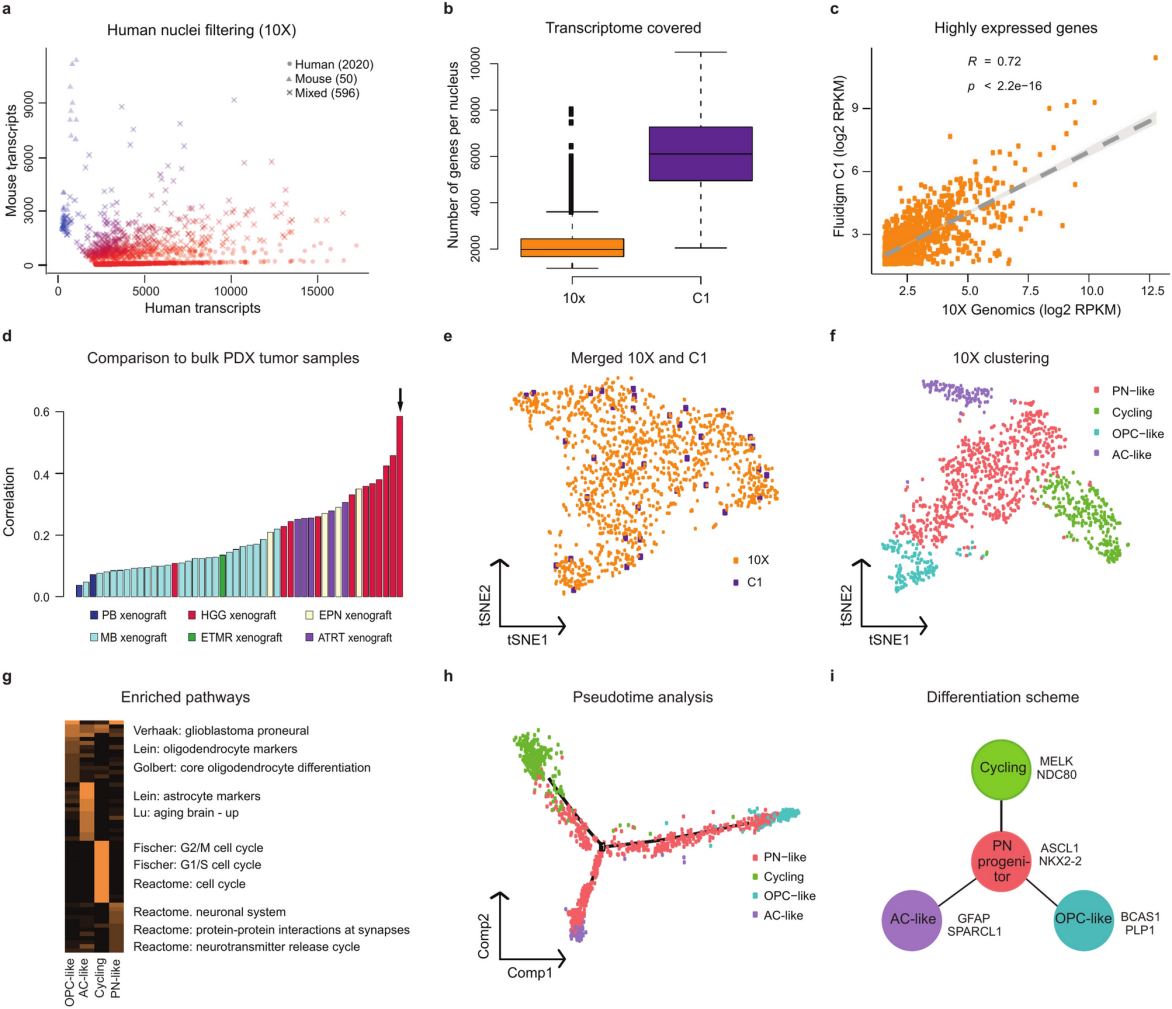
Comparison of platforms for studying tumor heterogeneity using patient-derived xenografts. (**a**) Variance between the numbers of human and mouse transcripts per nuclei from 10X snRNA- seq data of a glioma PDX sample. (**b**) Number of genes per cell and (**c**) gene expression levels between 10X and C1 platforms. (**d**) Comparison to bulk PDX tumor samples. 10X snRNA-seq data of the pHGG PDX sample combined into a pseudo-bulk compared to to bulk microarray data from a group of PDX samples based on correlation. The bulk microarray data of the same sample had the highest correlation to the 10X pseudo-bulk data (marked with an arrow). (**e**) *t*-distributed stochastic neighbour embedding (t-SNE) representation of combined 10X and C1 snRNA-seq datasets. (**f**) t-SNE representation of 10X PDX dataset. (**g**) Heatmap of pathways enriched among 10X PDX cell types. Colors represent confidence level –log10 (p-val). (**h**) Pseudotime trajectory analysis of 10X PDX cells. (i) Schematic representation of the identified tumor cell populations. See also Supplementary Fig. S2 and Supplementary Tables S2, S3, S4 and S5 online.

Separation of mouse and human nuclei was clear with both droplet-based as well as plate-based methods, with few mixed signals (Fig. 2a, Supplementary Fig. S2b-c online). Although the Fluidigm C1 analysis resulted in a much higher overall detection of genes per nucleus (~ 6000) compared with the droplet-based methods (10X Genomics ~ 2000 and Drop-seq ~ 1000) (Fig. 2b, Supplementary Fig. S2 d online, Supplementary Table S3 online), the proportion of human nuclei detected in comparison with mouse nuclei was greatly reduced (Supplementary Fig. S2e online). This difference may derive from physical differences of healthy mouse nuclei in comparison to tumorous human nuclei, and/or differences in nucleus size affecting capture efficiency, resulting in a differential flow in the C1 chip (although the results are based on small numbers overall). The poorer human to mouse nuclei ratio is also reflected in the presence of total human genes (Supplementary Fig. S2f. online). Despite this difference, however, the most highly expressed genes were still similar between 10X and C1 (Fig. 2c, Supplementary Fig. S2 g-h online). When comparing our snRNA-seq data of the PDX material merged as a pseudo-bulk with whole-tissue Affymetrix gene expression array data from multiple different patient tumors, the highest similarity was with the matched sample from which the PDX was derived (Fig. 2d). The other closely similar tumors belonged to the same tumor entity (pediatric high-grade glioma), with unrelated pediatric brain tumors showing a lower overall similarity. The same result was seen with both the 10X Genomics and the Fluidigm C1 datasets (not shown).

Notably, more mitochondrial genes were found in the Drop-seq data compared with the two commercial platforms (Supplementary Fig. S2i online). The Fluidigm C1 platform had an additional washing step included, which might have resulted in an improved filtering of the mitochondria. A clear explanation for the difference between the two droplet-based methods was not apparent, but one hypothesis could be that the lysis buffer in the Drop-seq may lyse the mitochondrial wall more efficiently than the lysis buffer used in 10X Genomics. The processing time of the Drop-seq is also higher than that for the 10X Genomics system, which results in a longer incubation time of the sample in the lysis buffer and thus possibly enhanced lysis of mitochondrial walls.

Although the inherent design differences of the two platforms means that more nuclei can be analyzed with 10X Genomics compared with Fluidigm C1, all the identified cellular subpopulations were found in the data of both systems (Fig. 2e, Supplementary Fig. S2j online). Due to the clearer differences in cluster designation with increased nucleus number, however, all subsequent analyses were performed with data from the 10X platform. The initial clustering of the PDX sample resulted in seven possible clusters (Supplementary Fig. S2k online). When assigning a putative identity to these clusters based on known marker gene expression^11^ (Supplementary Fig. S2 l online, Supplementary Table S4 online), we found evidence for four different cell populations: cycling cells, proneural-like (PN-like) cells, astrocyte-like (AC-like) cells, and oligodendrocyte precursor-like (OPC-like) cells (Fig. 2f). This assignment of the cell populations was further supported by analysis of enriched pathways (Fig. 2g, Supplementary Table S5 online). Pseudotime analysis of the nuclei data indicated three cellular states (Supplementary Fig. S2 m online). Mapping of cellular identities onto the pseudotime trajectory based on expression of known marker genes (Supplementary Fig. S2n-o online), suggested a model in which a cycling subpopulation gave rise to an intermediate PN-like progenitor (consisting of mesenchymal-like and neural progenitor-like cells) that could have subsequently differentiated into AC-like or OPC-like daughter cells (Fig. 2h-i).

### Single-nucleus RNA-seq of fresh frozen primary tumor tissues revealed distinct tumor and healthy cell populations

Nuclei from four long-term frozen primary pediatric glioma tissues (Supplementary Table S2 online) were analyzed using the 10X snRNA-seq system, which provided high-quality data with good confidence of transcriptome mapping (Supplementary Table S3 online). To investigate whether different sequencing devices impact cluster detection, we applied single cDNA libraries on multiple sequencers. Two of the samples were sequenced on three different machines (Illumina NextSeq500, HiSeq4000 and NovaSeq6000), with the estimated number of nuclei detected not substantially differing between the platforms (Supplementary Table S3 online). As expected, based on their output, the read coverage with NextSeq500 was lower than for the HiSeq and NovaSeq (the optimal sequencing saturation was reached with approximately 100,000 detected reads per nucleus). This was compensated for by reduced pooling of samples, and the proportion of reads mapped to the genome/transcriptome showed that the general performance was similar across all sequencing devices. An option for an optimal workflow could be to first run a pool of approximately 6–8 libraries on NextSeq500 to obtain an estimation of nuclei numbers and then re-run the libraries on NovaSeq6000 to reach sequencing saturation (more cost effective given the high number of reads required).

**Fig. 3:**
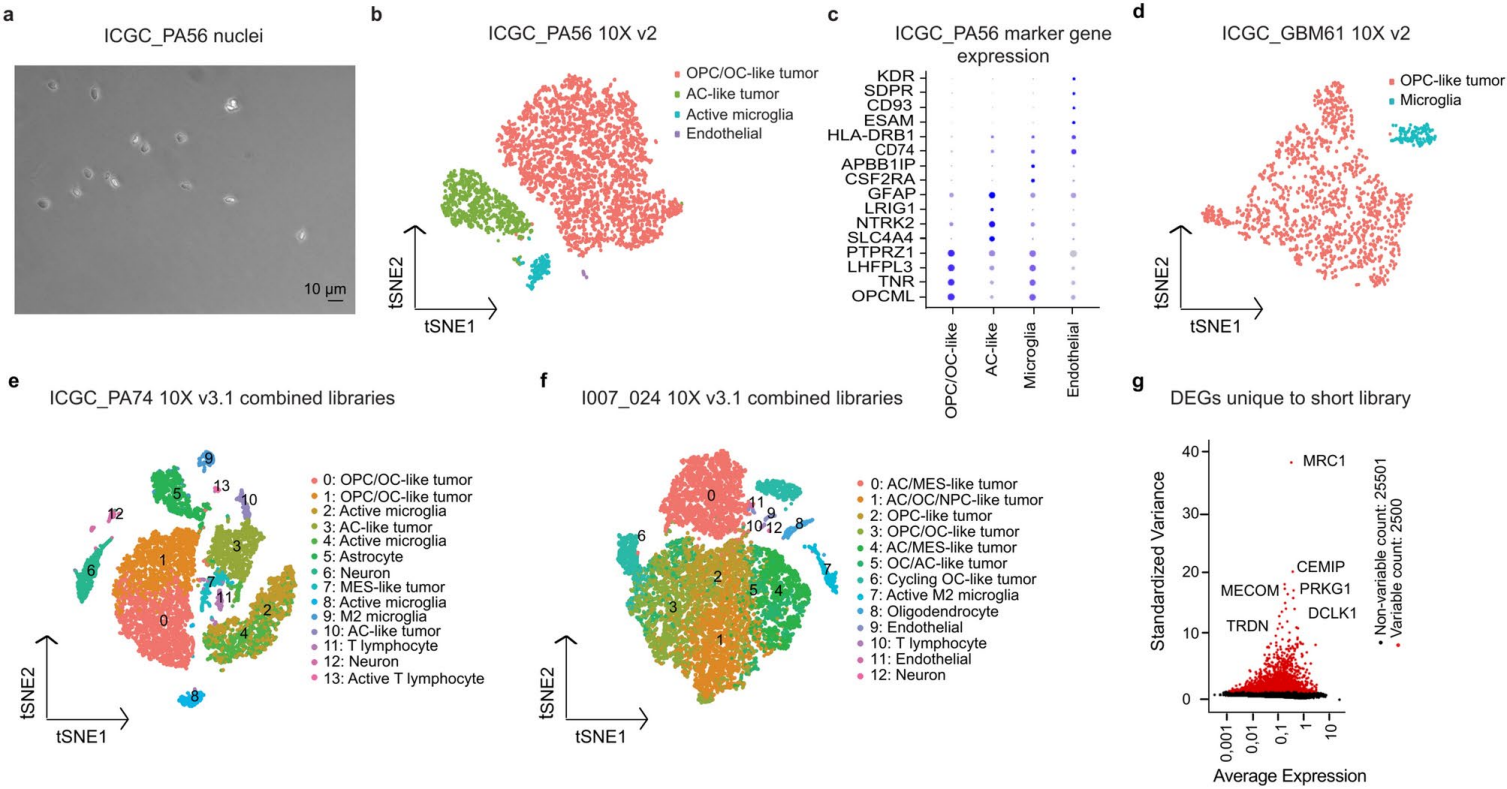
SnRNA-seq of fresh frozen primary tumor tissues reveal distinct tumor and healthy cell populations. (**a**) Brightfield image showing intact isolated nuclei loaded to the 10X system (scale bar 10 μm). (**b**) t-SNE representation of pilocytic astrocytoma ICGC_PA56 snRNA-seq data. Two tumor clusters, oligodendrocyte precursor/oligodendrocyte -like (OPC/OC-like) and astrocyte-like (AC-like) tumor cells clearly separated from microglia and endothelial cells. (**c**) The most highly expressed marker genes show differential expression across assigned cell types. (**d**) OPC-like tumor cells and microglia form separate clusters detected from glioblastoma ICGC_GBM61 snRNA-seq data. 10X v3.1 snRNA-seq data of combined libraries of standard (approx. 300–1000 bp) and short (< 400 bp) cDNA fragments gives a more detailed clustering of the tumors, here (**e**) pilocytic astrocytoma ICGC_PA74 and (**f**) pleomorphic xanthoastrocytoma I007_024. (**g**) Some genes are detected only from the short library data, here ICGC_PA74 as example. See also Supplementary Fig. S3 and S4 and Supplementary Tables S2, S3, S4 and S5 online.

The first tumor examined was a pilocytic astrocytoma sample (PA, a CNS WHO grade 1 tumor representing the most common childhood brain tumor) using snRNA-seq with multiple sequencing devices. The nuclei preparation was of good quality, despite a tissue storage time of 6 years (Fig. 3a). Overall cluster detection was similar with each of the sequencers (Supplementary Fig. S3a-c online), with some minor differences in cluster assignment of individual nuclei between platforms (Supplementary Fig. S3 d online). The clearest picture was obtained when combining all the data, confirming that a sufficient overall sequencing depth is more important for the identification of distinct cell populations than the specific device used to generate the data (Supplementary Fig. S3e online).

When looking in detail at this tumor (ICGC_PA56, harboring a *KIAA1549::BRAF*fusion – the most common genetic alteration in this entity^38^), a number of distinct cell types could be observed (Fig. 3b). Based on previously published marker genes for gliomas (see Materials & Methods), the largest tumor cell cluster was assigned as OPC/oligodendrocyte (OC) -like tumor cells (Fig. 3b-c), which have been suggested to be the cells-of-origin of PA tumors^39^. The cluster was also found to express genes associated with an active MAPK pathway (e.g., TRIO^40^, KCNQ1OT1^41^, and HIP1R^42^), further supporting a key role for MAPK-activated, OPC-derived tumor cells as a main component of PAs^43^. The other major cell population in ICGC_PA56 was identified to be AC-like tumor cells based on marker genes’ expression (Fig. 3b-c). These cells did not, however, uniformly resemble normal, healthy astrocytes when looked at highly expressed genes (Supplementary Table S4 online) using NeuroExpresso^44^and Brain RNA-seq^45^ databases, but showed instead a mix of multiple cell type marker genes. This indicates that they most likely represent a second (more differentiated) tumor cell subpopulation. Two putative healthy cell populations (active microglia and endothelial) were also identified within the PA sample (Fig. 3b-c). In contrast to the tumor cells, which often gave no clear match, these populations gave a relatively uniform result when comparing with NeuroExpresso and the Brain RNA-seq database as matching to microglia. These tools thus proved valuable for confirming the identity of clusters that may be otherwise difficult to assign.

The second tumor examined was a pediatric high-grade glioma (HGG), one of the most lethal forms of pediatric brain tumor with a typical survival of less than 2 years after diagnosis^46^. Analysis of the single nuclei data suggested two predominant clusters. Based on previously reported marker genes for glioma tumor cell populations (see Materials & Methods) the major cluster in this sample (ICGC_GBM61, as reported in^38,47^) seems to consist of OPC-like tumor cells (Fig. 3d). The additional second cluster was observed to be microglia, that was also confirmed from automatic comparison to existing reference.

When combining the snRNA-seq data of the low- and high-grade glioma samples, the non-tumor cells in common between the two were found to cluster more closely to each other than to other cells from the respective tumors, while the two tumor clusters were clearly distinct (Supplementary Fig. S4a online). This combined analysis confirmed the presence of microglia in both tumors. Despite their overall similarities, however, the immune cells also showed some differences (Supplementary Fig. S4b online), possibly hinting at different types or activation states of the microglia present. Further examination of these differences in future may provide information on, for example, tumor-promoting versus tumor- suppressing immune programs.

We further examined whether also including shorter cDNA fragments, which are usually removed from the standard 10X library, could provide useful additional information on single cell expression profiles. For that purpose, we analyzed two more pediatric glioma tissues, one additional PA (ICGC_PA74, *KIAA1549::BRAF* fusion) and one pleomorphic xanthoastrocytoma (PXA, a CNS WHO grade 2–3 tumor, I007_024, *BRAF V600E* and *CDK2 NA/B* deletion) (Supplementary Table S2 online). Distinct cell type clustering was very clear with these two samples, giving a good basis for manual and automatic cell type annotation from the combined short and standard libraries (see Materials & Methods).

The PA sample consisted of a major OPC/OC-like tumor cell population, a smaller AC-like tumor cell population and of multiple healthy cell populations (Fig. 3e). The PXA however, had a bigger tumor cluster, having characteristics of various cell types, such as OPC/OC-like tumors, AC-like tumors, and neural progenitor cell (NPC) -like tumor cells as well as mesenchymal (MES) -like tumor cells. There were, however, also healthy cell clusters detected, but clearly less than in the PA sample, which is expected considering the different grade of the tumors (Fig. 3f). The cell type assignment of SingleR with human healthy cerebellum reference gave a fairly similar result as the assignment based on marker gene lists (Supplementary Fig. S4e online). Interestingly, all the microglial activation genes that were previously postulated to be depleted in snRNA-seq datasets (*SPP1, CD74, FTL, APOE, FTH1, CST3, RPL29* and *APOC1*^48^) were found as differentially expressed genes in our snRNA-seq dataset (see ICGC_PA74 standard on Supplementary Table S4 online).

Both short and standard RNA fractions were congruent when defining clusters (Supplementary Fig. S4c-d online), but the short library gave a stronger verification for some cell types within clusters, e.g., defining M1 (inflammatory) and M2 (anti-inflammatory) microglial subtypes (Supplementary Fig. S4 d online). The microglia clusters (2 and 3) of ICGC_PA74 contained 2463 differentially expressed genes, of which 80 were unique to the short RNA fraction. The most highly variable unique gene for example, *MRC1*, is a marker for border-associated macrophages^49^ (Fig. 3g). There were at least 37 genes and 210 transcripts shorter than 400 bp (min. 189 bp and 186 bp, respectively)^50^ which could not be detected from a standard cDNA library. One such example is a protein coding gene *FAM25G* with a transcript length of 335 bp, which is differentially expressed in the ICGC_PA74 short library. Overall, we saw a clear benefit of adding an additional short-fragment library into the combined analysis of our snRNA-Seq tumor samples.

### Copy number variation analysis supports tumor cell detection from 10 × snRNA-seq data

**Fig. 4:**
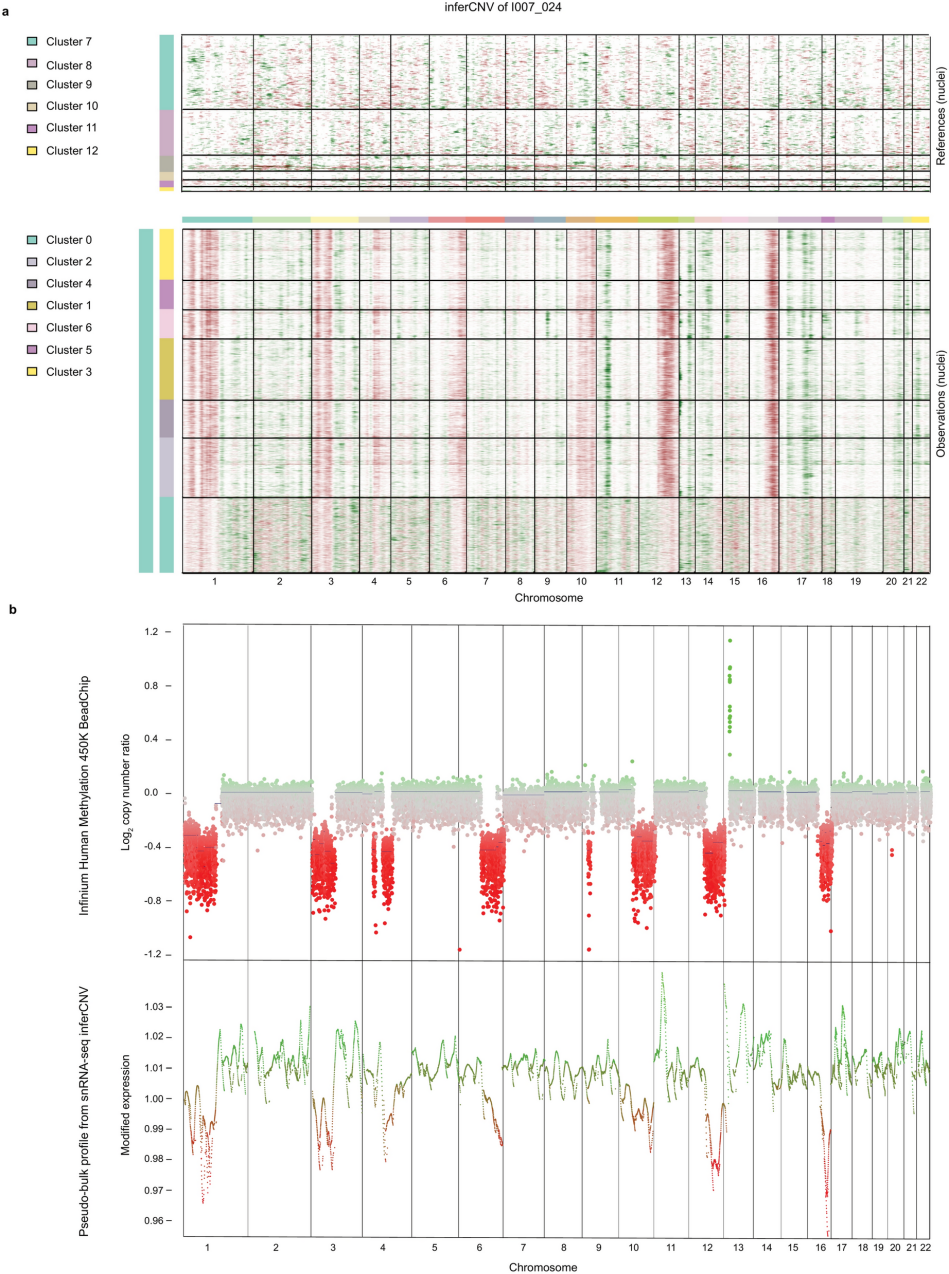
Copy number variation analysis supports tumor cell detection from 10 × snRNA- seq data. (**a**) Copy number variations (CNVs) of single nuclei from 10X snRNA-seq data of I007_024 analyzed by inferCNV. Non-malignant cells are used as control (upper heatmap). (**b**) A bulk CNV profile of the same tumor derived from Infinium HumanMethylation450 array analysis compared to pseudo-bulk extraction of CNV profiles from 10X data of I007_024 using mean values across the cells. See also Supplementary Fig. S5 and Supplementary Table S4 online.

Tools identifying tumor cell populations based on copy number variations (CNVs) have been developed for whole-transcriptome scRNA-seq methods such as the Fluidigm C1 or Smart-seq2 approaches^51,52^. The 10X Genomics system, however, amplifies only the 3’ ends of transcripts during preparation of the cDNA library. The total number of transcripts detected is also typically lower, making expression-based estimates of genomic copy number challenging. There are, however, methods being developed that also appear to be applicable to 10X data. One such tool for assessing copy number changes in tumor cell clusters from 10X snRNA-seq, inferCNV^53^, gave promising results despite technical limitations of 3’ read data (Fig. 4a and Supplementary Fig. S5a online). The healthy cell type clusters of I007_024 and ICGC_GBM61 were used as internal references for comparison with the tumor clusters of the same sample, with clear indications for regions of copy number change observed. While the overlap with the bulk CNV plot of the same tumor was limited overall, combining the single nucleus CNV results into pseudo-bulk demonstrated a reasonable correspondence for certain regions (Fig. 4b and Supplementary Fig. S5b online). For example, the calling of losses of chromosomes 1p, 3p, 4q, 6q, 10q, 12q and 16q and gains on chromosomes 11p, 13p and 17 withing I007_024 as well as loss of chromosome 16, gain on chromosome 6q and MYCN amplification within ICGC_GBM61, could potentially be used as an additional confirmation to annotate any ambiguous clusters as representing either tumor or stromal cells.

## Discussion

Here we have developed a method for extracting intact single nuclei from long-term frozen tissues. The method is simple, fast, low-cost, and is suitable for most typically equipped biology laboratories. The short processing time potentially reduces the risk of RNA degradation compared with some other protocols, and the method results in nuclei preparation with a good balance between purity and yield.

Previously, few protocols for using nuclei as scRNA-seq starting material have been published^20–27^. While some of these only features detection of few high quality cells using labor intensive protocols^20,21^, some more recent reports highlighted detection of multiple cell types, significantly increasing the utility^20–27^. Compared to these previous reports, the nucleus isolation presented here is tailored for isolating nuclei from frozen brain tumor samples, utilizes gentle douncing instead of mincing with scissors^22–26^ and provides an faster protocol resulting in high-quality data.

We found that nuclei extracted with our method can be applied to different scRNA-seq platforms. Both a full transcriptome amplification method (Fluidigm C1) and a 3’ amplification method (10X Genomics) proved suitable for the analysis of nuclei isolated from frozen glioma tissues, with minor differences in results deriving from general design characteristics of the platforms. The 3’ transcriptome amplification method, for example, detected fewer genes per nuclei (although this could also be partly linked to lower read-count per cell, and will likely improve with upcoming chemistry developments) but more nuclei in total, which lead to more distinct clustering and a more accurate distribution of human and mouse cell proportions. Due to the inherent design of the 3’ amplification method, genomic mutations could not be detected, but inferCNV analysis did allow a broad detection of copy number variations. The application to PDX samples may also be of interest in future, for example for unambiguously examining contributions of tumor (human) vs stromal (mouse) cell types to the overall signaling milieu in the bulk tissue.

Our study supports previous findings^10,21,54^ that nuclei from frozen tissues are a robust input material for single-cell omics analyses, thereby extending the range of suitable amenable to these high-resolution profiling techniques. This also removes the need to sort for viable whole cells, which possibly reduces sources of technical variation in the analyzed tissues. A bias for certain cell populations based on differential sensitivity to the mechanical forces or lysis buffers applied in this protocol, however, cannot be excluded at present. Thus, it must be kept in mind that the detected nuclei populations might differ quantitatively (and possibly also qualitatively) from the original cell composition.

Applying this method to pediatric brain tumor tissues also showed its potential for revealing biological insights. We were able to detect distinct tumor cell subpopulations in both low- and high-grade glioma samples and identified infiltrating immune cell components in all the tumors. Also, different activation states of the microglia were detected, as well as microglia with tumor signature, which may give a hint of the tumor supporting role of the immune cells. In addition, some tumor clusters were seen to be more mitotically active as others. The assignment of the cell type and the biological nature of the cluster was further enhanced with an inclusion of short (< 400 pb) fragments in the 10X RNA-library, which could give more insights especially into immune cell markers and transcription factors for example.

As conclusion, the possibility to extract and use nuclei from long-term frozen tissue material with a quick and simple protocol opens up enormous resources for the study of tumor heterogeneity and other questions of biological interest, expanding the utility of this rapidly developing method. This protocol is already in practice in multiple laboratories and successfully used in many studies, from which two are already published^55,56^.

## Methods

### Experimental model and subject details

*Human Subjects and Ethical Considerations:* Freshly frozen tissue biopsies were obtained from cerebellar and cortical brain areas from 4 pediatric glioma patients. The tissue biopsies were frozen in liquid nitrogen as such in plastic tubes without an embedding matrix. The patients were aged 15, 9 and 7 (male), and 7 and 14 (female). Patient metadata (age, gender, tumor location, diagnosis, and genetic alterations) are represented in Supplementary Table S2 online. Informed consent was obtained from patients and/or parents or legal guardians according to International Cancer Genome Consortium (ICGC) guidelines for all of the human tumor tissues used in this study. The ICGC study was approved by the Ethics Board of the Medical Faculty of the University of Heidelberg (S- 254/2009). All methods were conducted in accordance with all relevant guidelines and regulations.

*Patient-Derived Xenograft (PDX):* The PDX mouse used in this study was handled in accordance with legal and ethical regulations and approved by the regional council (Regierungspräsidium Karlsruhe, Germany; G- 64/14). Full details of the study according to ARRIVE guidelines can be found in Brabetz et al.^57^.

### Optimized nuclear extraction from frozen tumors

All surfaces were cleaned with RNAse Zap (Invitrogen AM9780) and PCR Clean wipes (Minerva Biolabs 15–2001) prior to sample processing, and equipment were cooled on ice and coated (0.1% Triton X- 100 [Sigma-Aldrich 93443] in filtered PBS (Merck Millipore SLGS033SS and Gibco 14,190–094). Only pipette tips of 1 ml (Biozym Low binding SafeSeal tips) and smaller were used to avoid losing the nuclei on the pipette tip walls.

A fresh frozen human brain tumor tissue piece (20–50 mg) was placed on a Petri dish (Greiner Bio-One 628,160) on a cooled metal block. It was mechanically dissociated using a scalpel in 1 ml of lysis buffer (0.32 M sucrose [Sigma-Aldrich 84097], 5 mM calcium dichloride [Sigma-Aldrich 21115], 3 mM magnesium acetate [Sigma-Aldrich 63052], 2.0 mM EDTA [Invitrogen 15,575–038], 0.5 mM EGTA [Alfa Aesar J61721], 10 mM Tris–HCl, pH 8.0 [Invitrogen AM98556], 1 mM DTT [Sigma-Aldrich 10197777001] and 0.1% Triton X- 100 [Sigma-Aldrich 93443]). An additional 4 ml of lysis buffer were added, and the tissue was dissociated further by pipetting and then transferred into a glass douncer (Sigma-Aldrich D9063). The cells were mechanically lysed by douncing 10 strokes with pestle A and then 10 strokes with pestle B. The lysate was directly filtered using a 100 μm filter (Greiner Bio-One 542,000) followed by a 40 μm filter (Greiner Bio-One 542,040) to remove the bigger cell membrane debris and then spun down to remove the lysis buffer (500 g, 5 min, 4 °C). The nuclei were re-suspended in 5 ml nuclei washing buffer (0.32 M sucrose [Sigma-Aldrich 84097], 5 mM calcium dichloride [Sigma-Aldrich 21115], 3 mM magnesium acetate [Sigma-Aldrich 63052], 2.0 mM EDTA [Invitrogen 15,575–038], 0.5 mM EGTA [Alfa Aesar J61721] and 10 mM Tris–HCl, pH 8.0 [Invitrogen AM98556]) in a 50 ml tube (Corning 352,070). The debris and leaking RNA were removed by centrifugation (500–600 g, 5 min, 4 °C). The washing steps should be done optimally two to three times (more washing rounds result in broken nuclear membranes). After the washing steps the nuclear pellet was re-suspended in about 50 μl – 1 ml of nuclei storage buffer (0.43 M sucrose [Sigma-Aldrich 84097], 70 mM potassium chloride [ThermoFischer Scientific, AM9640G], 2 mM magnesium dichloride [ThermoFischer Scientific AM95306], 10 mM Tris- HCl, pH 7.2 [Sigma-Aldrich T2069] and 5 mM EGTA [Alfa Aesar J61721]) (Supplementary Table S6 online).

When re-suspending the final nuclei preparation into the storage buffer, one should rather aim at too highly concentrated sample in regards of the optimal loading concentration required by the preferred single-cell platform, as it can always be diluted, but concentrating the final nucleus sample is likely to damage the nuclear membrane. If the nuclei pellet is only a smear on the tube wall, it is advised to use only 2 ml of washing buffer and after centrifugation, to only remove 1,5 ml of supernatant to avoid losing too many nuclei. In the cases of very small samples, instead of a second washing step, the remaining 500 μl could be centrifuged again, after which supernatant can be removed and the pellet resuspended in the storage buffer. If the starting tissue material weights less than 20 mg, it is advisable to reduce buffer volumes and use a smaller glass douncer. The nuclei can be processed directly in snRNA-seq platforms in the storage buffer or frozen shortly (a few days) at − 80 °C.

If the starting material for nuclear extraction is a frozen cell pellet, the mechanical lysis, cutting with scalpel, can be skipped. In case the pellet is very small, also filtering with 100 μm filter can be jumped over. The washing steps are the best to do with a volume of 3 ml and use 15 ml tubes instead of 50 ml ones. The optimal number of washes for pellets is 1–2 rounds.

### Comparison to other nuclear extraction methods

The final method was developed based on the density gradient centrifugation protocol originally developed by Spalding et. al. (2005)^32^and modified by Ernst et. al. (2014)^33^. The optimized protocol was compared to three commercial methods in addition to the original density gradient centrifugation (sucrose cushion) method mentioned above using 40 mg of a frozen pilocytic astrocytoma tissue ICGC_PA105 in each (Supplementary Fig. S1 online). The commercial methods were Nuclei EZ Prep (Sigma-Aldrich, NUC101 - 1 KT), Isolation of Nuclei for Single-Cell RNA Sequencing (10X Genomics)^34^and OptiPrepTM^35^ and were carried out following the manufacturer’s instructions. The final re-suspension volumes were as follows: 1 ml for our optimized protocol, 200 μl for Nuclei EZ Prep, 500 μl for the 10X protocol, and for OptiPrep and the sucrose cushion method, a 500 μl fraction was taken. OptiPrepTM was used as an alternative density gradient centrifugation method and applied after cell lysis and filtering. The nuclear preparations were observed for quality using light microscopy (Supplementary Fig. S1 A online).

### Nuclei staining experiments and microscopy

To test the optimal number of washing steps, 37 mg each of three different frozen glioma samples were processed using the optimized nuclear isolation protocol. Each sample was washed four times after nuclear isolation. After each wash, the nuclear pellet was re-suspended in 1 ml of storage buffer and a 50 μl sample taken. A further 4 ml of washing buffer was added before continuing to the next washing step. The nuclei were counted using an automated cell counter (Fig. 1C) and imaged using a light microscope (Zeiss Cell Observer) (Fig. 1B).

To study the condition of the isolated nuclei and possible RNA leakage, the nuclei were stained using DNA, RNA and membrane stains. Nuclei were isolated from frozen pediatric pilocytic astrocytoma tumor tissue using the optimized protocol with three washing steps. The nuclei were stored at − 80 °C overnight. On the next day, the nuclei were stained with RNA stain (SYTO RNASelect™ Green Fluorescent cell Stain [ThermoFischer Scientific, S32703]), DNA stain (Hoechst 33,342 Solution [ThermoFischer Scientific, 62249]) and membrane stain (BODIPYTM TR Ceramide [ThermoFischer Scientific, D7540]) and imaged using a fluorescent microscope (Olympus IX71) (Fig. 1D).

### SnRNA-seq experiments

Libraries from single nuclei were generated using either Chromium 10X Genomics^29^, Fluidigm C1^31^or Drop-seq^30^ platforms according to the manufacturers’ instructions as described in Macosko et al^30^ and Bageritz et al^36^ (for Drop-seq). 10 000 nuclei were loaded into 10X v2 and 20 000 into 10X v3.1 A 96-well plate was used for C1 and an estimated 2000 beads were exposed to a nuclei in Drop-seq. For Drop-seq, nuclei concentration was adjusted to the smaller droplets to not show more than 5% doublets. The PDX sample was loaded freshly after nuclear isolation into each snRNA-seq system, and the primary human tumor nuclei (ICGC_PA56 and ICGC_GBM61) were frozen overnight at − 80 C prior to 10X v2 experiments. Single Cell 3’ Reagent Kit v2 and v3.1 were used for 10X while SMARTer Ultra Low RNA Kit for the Fluidigm C1 System (small-cell IFCs). Two cDNA libraries were constructed per 10X v3.1 sample (ICGC_PA74 and I007_024): a standard cDNA library with double sided size selection and a library consisting only the short cDNA fragments, which was created using short cDNA fragments (approx. 400 bp) that would have otherwise been discarded in the second size selection process (these two libraries were then computationally combined in the data analysis). After the cDNA library construction, all the 10X v2 samples were sequenced on a HiSeq4000 sequencer (Illumina), and some of the samples (Supplementary Table S3 online) using NextSeq 500 (Illumina) or NovaSeq6000 (Illumina) (paired end 26 + 74 bp, single index 8 bp). The 10X v3.1 libraries were sequenced on a NovaSeq6000 (paired end 28 + 90 bp, dual index 10 bp), the C1 libraries on a HiSeq2000 (50 bp single-end dual index 2 × 8 bp) and Drop-seq libraries on a HiSeq2500 (paired end 20 + 180 bp, 8 bp single index).

### Bulk sequencing experiments

RNA from frozen tumor bulk tissues was used for Affymetrix gene expression array (Affymetrix Human Genome U133 Plus 2.0) and DNA in Infinium Methylation EPIC kit (Illumina).

### SnRNA-Seq data analysis

The initial processing of sequencing reads was performed independently for each platform. For 10X data the original CellRanger v2.1.0 pipeline was applied with an intron-including genome reference. Fluidigm C1 read alignment was performed with STAR v2.5.2b^58^to the genome reference combined with ERCC and gene expression counts computed using featureCounts v1.4.6^59^. Drop-seq reads were processed using Drop-seq tools v1.12^30^. For the alignment of PDX samples, combined hg19/mm10 reference was used, while tumor sample processing was performed with hg19 only. For PDX samples the differentiation between human and mouse cells was performed based on the comparison of transcript counts per species. The filtering cut limit for each platform was selected based on computation of the mean proportion of human genes per cell. The cells were assigned as human when the proportion of unique molecular identifiers (UMIs) covering human genes was more than 90%, and similarly as mice when the proportion of UMIs covering mouse genes was more than 90%. If none of these requirements was met, the cell was assigned as mixed.

Quality control was performed for each sample using Scater package v1.8.0^60^. For 10X and Drop-seq samples, cells with less than 500 genes and cells with a mitochondrial proportion higher than 1%, were excluded, approximately 10% of the cells were filtered out. For C1 samples, the minimum gene number per cell was 1500 and maximum mitochondrial proportion 1%. Approximately 3% of the cells processed with C1 were excluded. The dimension reduction, *t*-distributed stochastic neighbor embedding (tSNE) visualization, cell clustering and differential expression analysis for PDX 10X data was achieved using the Seurat v2.3.4^61^, while for the tumor samples with v3.2.2. The following Seurat functions were used: NormalizeData, FindVariableGenes with amount of 2500, ScaleData (number of UMI, mitochondrial), 10 principal components used for tSNE and uniform manifold approximation and projection (UMAP) visualization and Louvain algorithm for clustering. The integration of 10X and C1 data for the PDX sample was performed using Canonical Correlation Analysis (CCA) from Seurat. Trajectory reconstruction was achieved from the application of Monocle v2.8.0^62^on the adjusted result from Seurat. Cell type assignment was initially done by manually looking for marker genes specific for different normal cell types in the differentially expressed genes list using public cell type resources such as Brain RNA-seq^45^and NeuroExpresso^44^. Previously published gene lists derived from single-cell RNA-sequencing data of brain tumors^6,11,14,15,39,63^ were used to manually annotate clusters, which were not clearly annotated as any healthy cell type.

Correspondence of the cell types to specific pathways was also computed using hyper geometric test applied on functional gene lists from the Molecular Signatures Database (MSigDB) collection^64^. In addition, the assignment was verified by projecting to normal cerebellum cell types using SingleR. Combined visualization of cluster similarity between platforms was performed using GoogleVis R package with assignment based on a positive correlation limit of 0.3.

Copy number profiling was performed from the usage of inferCNV v1.3.2 of the Trinity CTAT Project as previously described^14^. Gene expression levels per nucleus were visualized using a custom analysis script kindly made available by Dr. Murat Iskar (unpublished).

### Affymetrix data integration

The Affymetrix PDX data was collected from Brabetz et al.^57^. Mean values between probes per gene were computed to generate a full normalized expression matrix. PDX single nucleus profiles (10X and C1) were combined into bulk datasets and normalized via Reads per kilobase of transcript per million mapped reads (RPKM) normalization method for the comparison with Affymetrix data. Correlation was computed based on the selection of the top 500 most highly variable genes in common between the snRNA-seq sample and the Affymetrix matrix.

### Quantification and statistical analysis

Statistical analyses were performed using custom R code based on the usage integrated packages and Prism (GraphPad) toolkit. In case of Prism  (Fig. 1C) data are represented as mean with standard error of the mean.

## Supplementary Information

Supplementary Information 1.

Supplementary Information 2.

Supplementary Information 3.

## Data Availability

The datasets generated during the current study are available in the GEO repository, https://www.ncbi.nlm.nih.gov/geo/query/acc.cgi?acc = GSE268496.

## References

[1] Filbin, M. & Monje, M. Developmental origins and emerging therapeutic opportunities for childhood cancer. *Nat. Med.* **25**, 367–376. 10.1038/s41591-019-0383-9 (2019). 30842674 10.1038/s41591-019-0383-9 PMC6631320

[2] Sturm, D., Pfister, S. M. & Jones, D. T. W. Pediatric gliomas: Current concepts on diagnosis, biology, and clinical management. *J. Clin. Oncol.* **35**, 2370–2377. 10.1200/JCO.2017.73.0242 (2017). 28640698 10.1200/JCO.2017.73.0242

[3] Liu, K. W., Pajtler, K. W., Worst, B. C., Pfister, S. M. & Wechsler-Reya, R. J. Molecular mechanisms and therapeutic targets in pediatric brain tumors. *Sci. Signal*10.1126/scisignal.aaf7593 (2017). 28292958 10.1126/scisignal.aaf7593

[4] Kallappagoudar, S., Yadav, R. K., Lowe, B. R. & Partridge, J. F. Histone H3 mutations–a special role for H3.3 in tumorigenesis?. *Chromosoma* **124**, 177–189. 10.1007/s00412-015-0510-4 (2015). 25773741 10.1007/s00412-015-0510-4 PMC4446520

[5] Danilenko, M. et al. Single-cell DNA sequencing identifies risk-associated clonal complexity and evolutionary trajectories in childhood medulloblastoma development. *Acta Neuropathol.* **144**, 565–578. 10.1007/s00401-022-02464-x (2022). 35831448 10.1007/s00401-022-02464-x PMC9381458

[6] Filbin, M. G. et al. Developmental and oncogenic programs in H3K27M gliomas dissected by single-cell RNA-seq. *Science* **360**, 331–335. 10.1126/science.aao4750 (2018). 29674595 10.1126/science.aao4750 PMC5949869

[7] Gillen, A. E. et al. Single-cell RNA sequencing of childhood ependymoma reveals neoplastic cell subpopulations that impact molecular classification and etiology. *Cell. Rep.* **32**, 108023. 10.1016/j.celrep.2020.108023 (2020). 32783945 10.1016/j.celrep.2020.108023 PMC7452755

[8] Gojo, J. et al. Single-cell RNA-seq reveals cellular hierarchies and impaired developmental trajectories in pediatric ependymoma. *Cancer Cell* **38**, 44-59.e49. 10.1016/j.ccell.2020.06.004 (2020). 32663469 10.1016/j.ccell.2020.06.004 PMC7479515

[9] Hovestadt, V. et al. Resolving medulloblastoma cellular architecture by single-cell genomics. *Nature* **572**, 74–79. 10.1038/s41586-019-1434-6 (2019). 31341285 10.1038/s41586-019-1434-6 PMC6754173

[10] Lake, B. B. et al. A comparative strategy for single-nucleus and single-cell transcriptomes confirms accuracy in predicted cell-type expression from nuclear RNA. *Sci. Rep.* **7**, 6031. 10.1038/s41598-017-04426-w (2017). 28729663 10.1038/s41598-017-04426-w PMC5519641

[11] Neftel, C. et al. An integrative model of cellular states, plasticity, and genetics for glioblastoma. *Cell* **178**, 835-849.e821. 10.1016/j.cell.2019.06.024 (2019). 31327527 10.1016/j.cell.2019.06.024 PMC6703186

[12] Pun, M. et al. Common molecular features of H3K27M DMGs and PFA ependymomas map to hindbrain developmental pathways. *Acta Neuropathol. Commun.* **11**, 25. 10.1186/s40478-023-01514-z (2023). 36759899 10.1186/s40478-023-01514-z PMC9912509

[13] Riemondy, K. A. et al. Neoplastic and immune single-cell transcriptomics define subgroup-specific intra-tumoral heterogeneity of childhood medulloblastoma. *Neuro. Oncol.* **24**, 273–286. 10.1093/neuonc/noab135 (2022). 34077540 10.1093/neuonc/noab135 PMC8804892

[14] Tirosh, I. et al. Single-cell RNA-seq supports a developmental hierarchy in human oligodendroglioma. *Nature* **539**, 309–313. 10.1038/nature20123 (2016). 27806376 10.1038/nature20123 PMC5465819

[15] Venteicher, A. S. et al. Decoupling genetics, lineages, and microenvironment in IDH-mutant gliomas by single-cell RNA-seq. *Science*10.1126/science.aai8478 (2017). 28360267 10.1126/science.aai8478 PMC5519096

[16] Wu, H. et al. Single-cell RNA sequencing unravels upregulation of immune cell crosstalk in relapsed pediatric ependymoma. *Front. Immunol.* **13**, 903246. 10.3389/fimmu.2022.903246 (2022). 35844565 10.3389/fimmu.2022.903246 PMC9281506

[17] Yuan, J. et al. Single-cell transcriptome analysis of lineage diversity in high-grade glioma. *Genome Med.* **10**, 57. 10.1186/s13073-018-0567-9 (2018). 30041684 10.1186/s13073-018-0567-9 PMC6058390

[18] Zhang, L. et al. Single-cell transcriptomics in medulloblastoma reveals tumor-initiating progenitors and oncogenic cascades during tumorigenesis and relapse. *Cancer Cell* **36**, 302-318.e307. 10.1016/j.ccell.2019.07.009 (2019). 31474569 10.1016/j.ccell.2019.07.009 PMC6760242

[19] Habib, N. et al. Div-Seq: Single-nucleus RNA-Seq reveals dynamics of rare adult newborn neurons. *Science* **353**, 925–928. 10.1126/science.aad7038 (2016). 27471252 10.1126/science.aad7038 PMC5480621

[20] Hu, P. et al. Dissecting cell-type composition and activity-dependent transcriptional state in mammalian brains by massively parallel single-nucleus RNA-seq. *Mol. Cell* **68**, 1006-1015.e1007. 10.1016/j.molcel.2017.11.017 (2017). 29220646 10.1016/j.molcel.2017.11.017 PMC5743496

[21] Bakken, T. E. et al. Single-nucleus and single-cell transcriptomes compared in matched cortical cell types. *PLoS ONE* **13**, e0209648. 10.1371/journal.pone.0209648 (2018). 30586455 10.1371/journal.pone.0209648 PMC6306246

[22] Slyper, M. et al. A single-cell and single-nucleus RNA-Seq toolbox for fresh and frozen human tumors. *Nat. Med.* **26**, 792–802. 10.1038/s41591-020-0844-1 (2020). 32405060 10.1038/s41591-020-0844-1 PMC7220853

[23] Ding, J. et al. Systematic comparison of single-cell and single-nucleus RNA-sequencing methods. *Nat. Biotechnol.* **38**, 737–746. 10.1038/s41587-020-0465-8 (2020). 32341560 10.1038/s41587-020-0465-8 PMC7289686

[24] Narayanan, A. et al. Nuclei isolation from fresh frozen brain tumors for single-nucleus RNA-seq and ATAC-seq. *J. Vis. Exp.*10.3791/61542 (2020). 32925882 10.3791/61542

[25] Wang, Y. et al. Multimodal single-cell and whole-genome sequencing of small, frozen clinical specimens. *Nat. Genet* **55**, 19–25. 10.1038/s41588-022-01268-9 (2023). 36624340 10.1038/s41588-022-01268-9 PMC10155259

[26] Liu, I. et al. The landscape of tumor cell states and spatial organization in H3–K27M mutant diffuse midline glioma across age and location. *Nat. Genet* **54**, 1881–1894. 10.1038/s41588-022-01236-3 (2022). 36471067 10.1038/s41588-022-01236-3 PMC9729116

[27] Lee, M. K. et al. Identifying tumor type and cell type-specific gene expression alterations in pediatric central nervous system tumors. *Nat. Commun.* **15**, 3634. 10.1038/s41467-024-47712-8 (2024). 38688897 10.1038/s41467-024-47712-8 PMC11061189

[28] Habib, N. et al. Massively parallel single-nucleus RNA-seq with DroNc-seq. *Nat. Methods* **14**, 955–958. 10.1038/nmeth.4407 (2017). 28846088 10.1038/nmeth.4407 PMC5623139

[29] Zheng, G. X. et al. Massively parallel digital transcriptional profiling of single cells. *Nat. Commun.* **8**, 14049. 10.1038/ncomms14049 (2017). 28091601 10.1038/ncomms14049 PMC5241818

[30] Macosko, E. Z. et al. Highly parallel genome-wide expression profiling of individual cells using nanoliter droplets. *Cell* **161**, 1202–1214. 10.1016/j.cell.2015.05.002 (2015). 26000488 10.1016/j.cell.2015.05.002 PMC4481139

[31] Lake, B. B. et al. Neuronal subtypes and diversity revealed by single-nucleus RNA sequencing of the human brain. *Science* **352**, 1586–1590. 10.1126/science.aaf1204 (2016). 27339989 10.1126/science.aaf1204 PMC5038589

[32] Spalding, K. L., Bhardwaj, R. D., Buchholz, B. A., Druid, H. & Frisén, J. Retrospective birth dating of cells in humans. *Cell* **122**, 133–143. 10.1016/j.cell.2005.04.028 (2005). 16009139 10.1016/j.cell.2005.04.028

[33] Ernst, A. et al. Neurogenesis in the striatum of the adult human brain. *Cell* **156**, 1072–1083. 10.1016/j.cell.2014.01.044 (2014). 24561062 10.1016/j.cell.2014.01.044

[34] 3410xGenomics. (2017).

[35] AxisShield. (2018).

[36] Bageritz, J. & Raddi, G. Single-cell RNA sequencing with drop-seq. *Methods Mol. Biol.* **1979**, 73–85. 10.1007/978-1-4939-9240-9_6 (2019). 31028633 10.1007/978-1-4939-9240-9_6

[37] Abdolahi, S. et al. Patient-derived xenograft (PDX) models, applications and challenges in cancer research. *J. Transl. Med.* **20**, 206. 10.1186/s12967-022-03405-8 (2022). 35538576 10.1186/s12967-022-03405-8 PMC9088152

[38] Jones, D. T. et al. Recurrent somatic alterations of FGFR1 and NTRK2 in pilocytic astrocytoma. *Nat. Genet* **45**, 927–932. 10.1038/ng.2682 (2013). 23817572 10.1038/ng.2682 PMC3951336

[39] Reitman, Z. J. et al. Mitogenic and progenitor gene programmes in single pilocytic astrocytoma cells. *Nat. Commun.* **10**, 3731. 10.1038/s41467-019-11493-2 (2019). 31427603 10.1038/s41467-019-11493-2 PMC6700116

[40] Bellanger, J. M. et al. The two guanine nucleotide exchange factor domains of Trio link the Rac1 and the RhoA pathways in vivo. *Oncogene* **16**, 147–152. 10.1038/sj.onc.1201532 (1998). 9464532 10.1038/sj.onc.1201532

[41] Wang, H. et al. LncRNA KCNQ1OT1 (potassium voltage-gated channel subfamily Q member 1 opposite strand/antisense transcript 1) aggravates acute kidney injury by activating p38/NF-κB pathway via miR-212-3p/MAPK1 (mitogen-activated protein kinase 1) axis in sepsis. *Bioengineered* **12**, 11353–11368. 10.1080/21655979.2021.2005987 (2021). 34783627 10.1080/21655979.2021.2005987 PMC8810185

[42] Yang, Q. et al. Endocytic adaptor protein HIP1R controls intracellular trafficking of epidermal growth factor receptor in neuronal dendritic development. *Front. Mol. Neurosci.* **11**, 447. 10.3389/fnmol.2018.00447 (2018). 30574069 10.3389/fnmol.2018.00447 PMC6291753

[43] Jones, D. T. W., Bandopadhayay, P. & Jabado, N. The power of human cancer genetics as revealed by low-grade gliomas. *Annu. Rev. Genet* **53**, 483–503. 10.1146/annurev-genet-120417-031642 (2019). 31794268 10.1146/annurev-genet-120417-031642

[44] Mancarci, B. O. et al. Cross-laboratory analysis of brain cell type transcriptomes with applications to interpretation of bulk tissue data. *eNeuro*10.1523/ENEURO.0212-17.2017 (2017). 29204516 10.1523/ENEURO.0212-17.2017 PMC5707795

[45] Zhang, Y. et al. An RNA-sequencing transcriptome and splicing database of glia, neurons, and vascular cells of the cerebral cortex. *J. Neurosci.* **34**, 11929–11947. 10.1523/JNEUROSCI.1860-14.2014 (2014). 25186741 10.1523/JNEUROSCI.1860-14.2014 PMC4152602

[46] Jones, C. et al. Pediatric high-grade glioma: Biologically and clinically in need of new thinking. *Neuro. Oncol.* **19**, 153–161. 10.1093/neuonc/now101 (2017). 27282398 10.1093/neuonc/now101 PMC5464243

[47] Project, I. C. G. C. P. T. Recurrent MET fusion genes represent a drug target in pediatric glioblastoma. *Nat. Med.* **22**, 1314–1320. 10.1038/nm.4204 (2016). 27748748 10.1038/nm.4204

[48] Thrupp, N. et al. Single-nucleus RNA-seq is not suitable for detection of microglial activation genes in humans. *Cell Rep.* **32**, 108189. 10.1016/j.celrep.2020.108189 (2020). 32997994 10.1016/j.celrep.2020.108189 PMC7527779

[49] Van Hove, H. et al. A single-cell atlas of mouse brain macrophages reveals unique transcriptional identities shaped by ontogeny and tissue environment. *Nat. Neurosci.* **22**, 1021–1035. 10.1038/s41593-019-0393-4 (2019). 31061494 10.1038/s41593-019-0393-4

[50] Piovesan, A. et al. Human protein-coding genes and gene feature statistics in 2019. *BMC Res. Notes* **12**, 315. 10.1186/s13104-019-4343-8 (2019). 31164174 10.1186/s13104-019-4343-8 PMC6549324

[51] Müller, S., Cho, A., Liu, S. J., Lim, D. A. & Diaz, A. CONICS integrates scRNA-seq with DNA sequencing to map gene expression to tumor sub-clones. *Bioinformatics* **34**, 3217–3219. 10.1093/bioinformatics/bty316 (2018). 29897414 10.1093/bioinformatics/bty316 PMC7190654

[52] Fan, J. et al. Linking transcriptional and genetic tumor heterogeneity through allele analysis of single-cell RNA-seq data. *Genome Res.* **28**, 1217–1227. 10.1101/gr.228080.117 (2018). 29898899 10.1101/gr.228080.117 PMC6071640

[53] Lake, B. B. et al. Integrative single-cell analysis of transcriptional and epigenetic states in the human adult brain. *Nat. Biotechnol.* **36**, 70–80. 10.1038/nbt.4038 (2018). 29227469 10.1038/nbt.4038 PMC5951394

[54] Grindberg, R. V. et al. RNA-sequencing from single nuclei. *Proc. Natl. Acad. Sci. U. S. A.* **110**, 19802–19807. 10.1073/pnas.1319700110 (2013). 24248345 10.1073/pnas.1319700110 PMC3856806

[55] Ghasemi, D. R. et al. Compartments in medulloblastoma with extensive nodularity are connected through differentiation along the granular precursor lineage. *Nat. Commun.* **15**, 269. 10.1038/s41467-023-44117-x (2024). 38191550 10.1038/s41467-023-44117-x PMC10774372

[56] Hai, L. et al. A clinically applicable connectivity signature for glioblastoma includes the tumor network driver CHI3L1. *Nat. Commun.* **15**, 968. 10.1038/s41467-024-45067-8 (2024). 38320988 10.1038/s41467-024-45067-8 PMC10847113

[57] Brabetz, S. et al. A biobank of patient-derived pediatric brain tumor models. *Nat. Med.* **24**, 1752–1761. 10.1038/s41591-018-0207-3 (2018). 30349086 10.1038/s41591-018-0207-3

[58] Dobin, A. et al. STAR: Ultrafast universal RNA-seq aligner. *Bioinformatics* **29**, 15–21. 10.1093/bioinformatics/bts635 (2013). 23104886 10.1093/bioinformatics/bts635 PMC3530905

[59] Liao, Y., Smyth, G. K. & Shi, W. featureCounts: An efficient general purpose program for assigning sequence reads to genomic features. *Bioinformatics* **30**, 923–930. 10.1093/bioinformatics/btt656 (2014). 24227677 10.1093/bioinformatics/btt656

[60] McCarthy, D. J., Campbell, K. R., Lun, A. T. & Wills, Q. F. Scater: Pre-processing, quality control, normalization and visualization of single-cell RNA-seq data in R. *Bioinformatics* **33**, 1179–1186. 10.1093/bioinformatics/btw777 (2017). 28088763 10.1093/bioinformatics/btw777 PMC5408845

[61] Butler, A., Hoffman, P., Smibert, P., Papalexi, E. & Satija, R. Integrating single-cell transcriptomic data across different conditions, technologies, and species. *Nat. Biotechnol.* **36**, 411–420. 10.1038/nbt.4096 (2018). 29608179 10.1038/nbt.4096 PMC6700744

[62] Qiu, X. et al. Reversed graph embedding resolves complex single-cell trajectories. *Nat. Methods* **14**, 979–982. 10.1038/nmeth.4402 (2017). 28825705 10.1038/nmeth.4402 PMC5764547

[63] Patel, A. P. et al. Single-cell RNA-seq highlights intratumoral heterogeneity in primary glioblastoma. *Science* **344**, 1396–1401. 10.1126/science.1254257 (2014). 24925914 10.1126/science.1254257 PMC4123637

[64] Liberzon, A. et al. The molecular signatures database (MSigDB) hallmark gene set collection. *Cell Syst.* **1**, 417–425. 10.1016/j.cels.2015.12.004 (2015). 26771021 10.1016/j.cels.2015.12.004 PMC4707969

