# Supplementary material for: A simplified preparation method for single-nucleus RNA-sequencing using long-term frozen brain tumor tissues
Source: Sci Rep. 2025 Apr 14;15:12849. doi: 10.1038/s41598-025-97053-9 (PMC11997191; doi:10.1038/s41598-025-97053-9)
Supplement: Supplementary file 1 — Supplementary Information 1. [file 41598_2025_97053_MOESM1_ESM.pdf]

Supplementary information

A simplified preparation method for single-nucleus RNA-sequencing using long-term frozen brain tumor tissues

Kati J. Ernst[1,2,8,] Konstantin Okonechnikov[1,3,8,] Josephine Bageritz[4], Ashwyn A. Perera[1,2,5], Jan-Philipp Mallm[6], Andrea Wittmann[1,2], Kendra K. Maaß[1,3], Svenja Leible[4], Michael Boutros[4], Stefan M. Pfister[1,3,7], Marc Zuckermann[1,3,9, *], David T.W. Jones[1,2,9*]

Supplementary Note 1. Protocol for isolation of nuclei from frozen glioma tissue/pellet

Prepare beforehand

- Clean surfaces with RNase Zap
- Pre-cool centrifuge (4°C) and buffers, douncer, cold plate and tubes on ice
- Coat all consumables (tubes, douncer, tips) just before use
- Use max. 1 ml pipette tips

Isolation from frozen tissue

- Take 5 ml of washing buffer and add 5 µl DTT (1M, stored at -20°C) and 50 µl Triton-X (10%) → lysis buffer
- Place the tissue on a petri dish on a cold plate
- Add 1 ml of lysis buffer and cut with a scalpel so that the tissue can be taken into a 1 ml pipette tip
- Add the rest 4 ml of lysis buffer and transfer into a douncer
- Dounce the suspension 10 times with pestle A and 10 times with pestle B
- Filter the entire mix with a 100 µm filter and then with a 40 µm filter
- Centrifuge 5 min with 500 g at 4°C
- Carefully remove supernatant with a pipette (1 ml) and discard
- Re-suspend the pellet in 2 ml of washing buffer, centrifuge 5 min with 600 g at 4°C and remove supernatant (if the pellet is large, re-suspend in 5 ml)
- Repeat the washing step 0-2 times (in total 1-3 washes)
- Re-suspend the nuclear pellet in storage buffer (depends on the pellet size, 50 µl – 1 ml)

Isolation from frozen cell pellet

- Prepare lysis buffer as described above
- Add the lysis buffer on the cell pellet, re-suspend and transfer into a douncer
- Dounce the suspension 10 times with pestle A and 10 times with pestle B
- Filter the entire mix with a 100 µm filter and then with a 40 µm filter
    - If the pellet is really small, 100 µm filter can be skipped
- Centrifuge 5 min with 500 g at 4°C
- Carefully remove supernatant with a pipette (1 ml) and discard
- Re-suspend the pellet in 2 ml of washing buffer and centrifuge 5 min with 600 g at 4°C, remove supernatant
- Repeat the washing step 0-1 times (in total 1-2 washes)
- Re-suspend the nuclear pellet in storage buffer (depends on the pellet size, 50 µl – 1 ml)

Cleaning the douncer

- Rinse with water and 70% ethanol and fill with 4 % natriumhypochloride
- Keep overnight in the hood
- Rinse again with water and autoclave

Washing Buffer

Chemical	Final concentration	Working volumes	Manufacturer
Sucrose	0.32 M	5.48 g	SIGMA 84097
$CaCl_2$	5 mM	250 µl	SIGMA 21115
Magnesium acetate $Mg(CH_3COO)_2$	3 mM	150 µl	SIGMA 63052
EDTA	2.0 mM	200 µl	Invitrogen 15575-038
EGTA	0.5 mM	50 µl	Alfa Aesar J61721
Tris-HCl (pH 8)	10 mM	500 µl	Invitrogen AM98556
Ultrapure H_2O		Until 50 ml	Invitrogen 10977023

Dissolve sucrose at 50°C (using a roller) first with about 30 ml water, then add the rest of the water until 50 ml

Lysis buffer

Chemical	Final concentration	Working volumes	Manufacturer
Washing buffer	1x	5 ml	
DTT	1 mM	5 µl	SIGMA 10197777001
Triton-X (10%)	0.1%	50 µl	SIGMA 93443

Add DTT and Triton-X always freshly before use

Nuclei Storage Buffer

Chemical	Final concentration	Working volumes	Manufacturer
Sucrose	0.43 M	7.36 g	SIGMA 84097
KCl	70 mM	1750 µl	Ambion AM9640G
$MgCl_2$	2 mM	100 µl	Invitrogen AM95306
EGTA	5 mM	500 µl	Alfa Aesar J61721
Tris-HCl (pH 7.2)	10 mM	500 µl	SIGMA T2069
Ultrapure H_2O		until 50 ml	Invitrogen 10977023

Dissolve Sucrose at 50°C

Coating Buffer

Chemical	Final concentration	Working volumes	Manufacturer
Triton-X (10%)	0.1%	500 µl	SIGMA 93443
DPBS (1x)	99.9%	50 ml	Invitrogen 14190094

Filter solution with a 0,22 µm filter

Buffers without DTT and Triton-X can be stored long-term at RT

Douncer: Sigma-Aldrich D9063

Supplementary figures

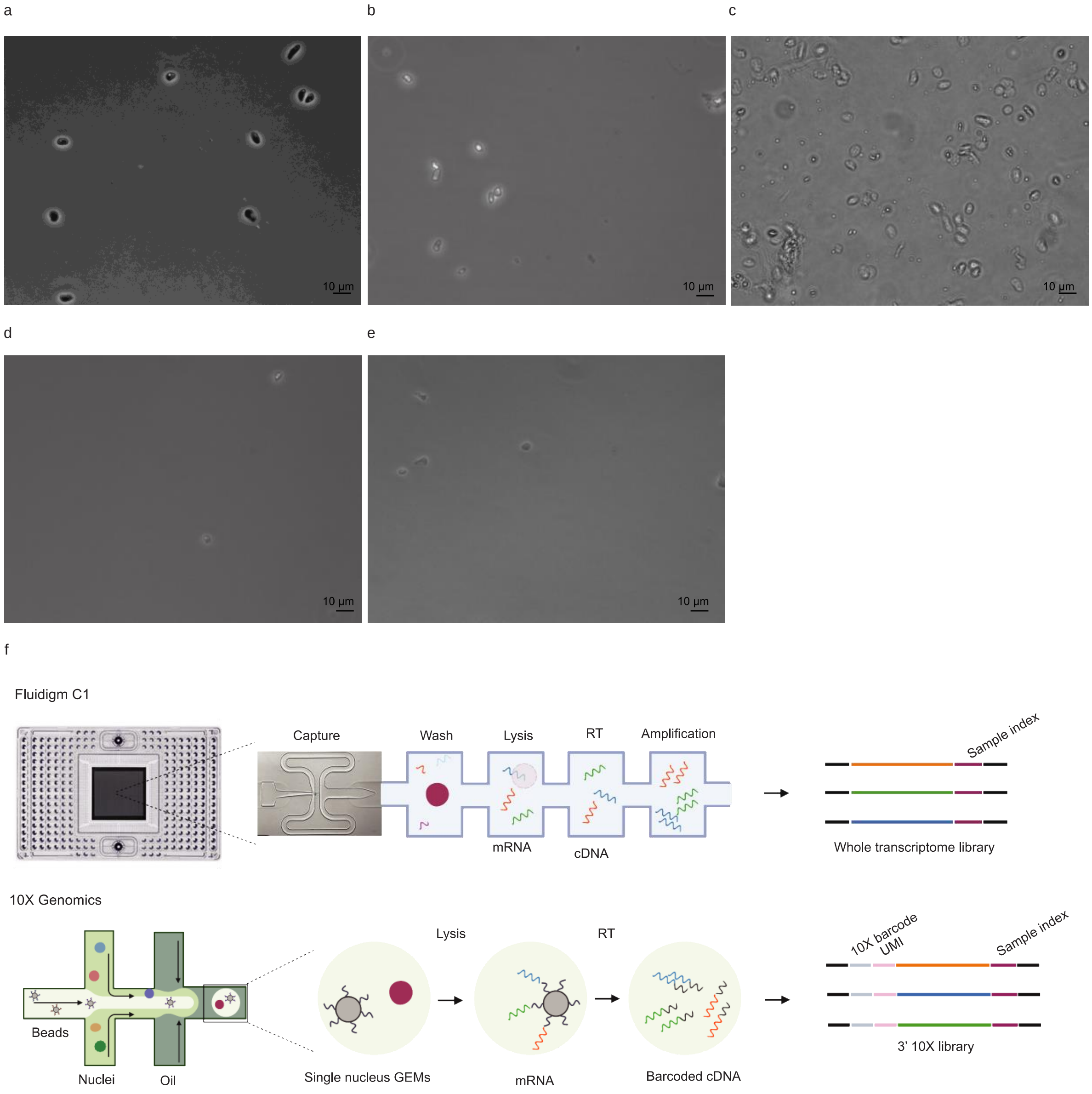

Figure S1. Comparison of the optimized nuclei isolation protocol to other protocols. Brightfield image of nuclei extracted from a pilocytic astrocytoma tissue using a) the optimized isolation protocol, b) sucrose cushion density gradient, c) Nuclei EZ Prep, d) Isolation of Nuclei for Single-Cell RNA Sequencing (10X Genomics), and e) OptiPrepTM (scale bar 10 µm).

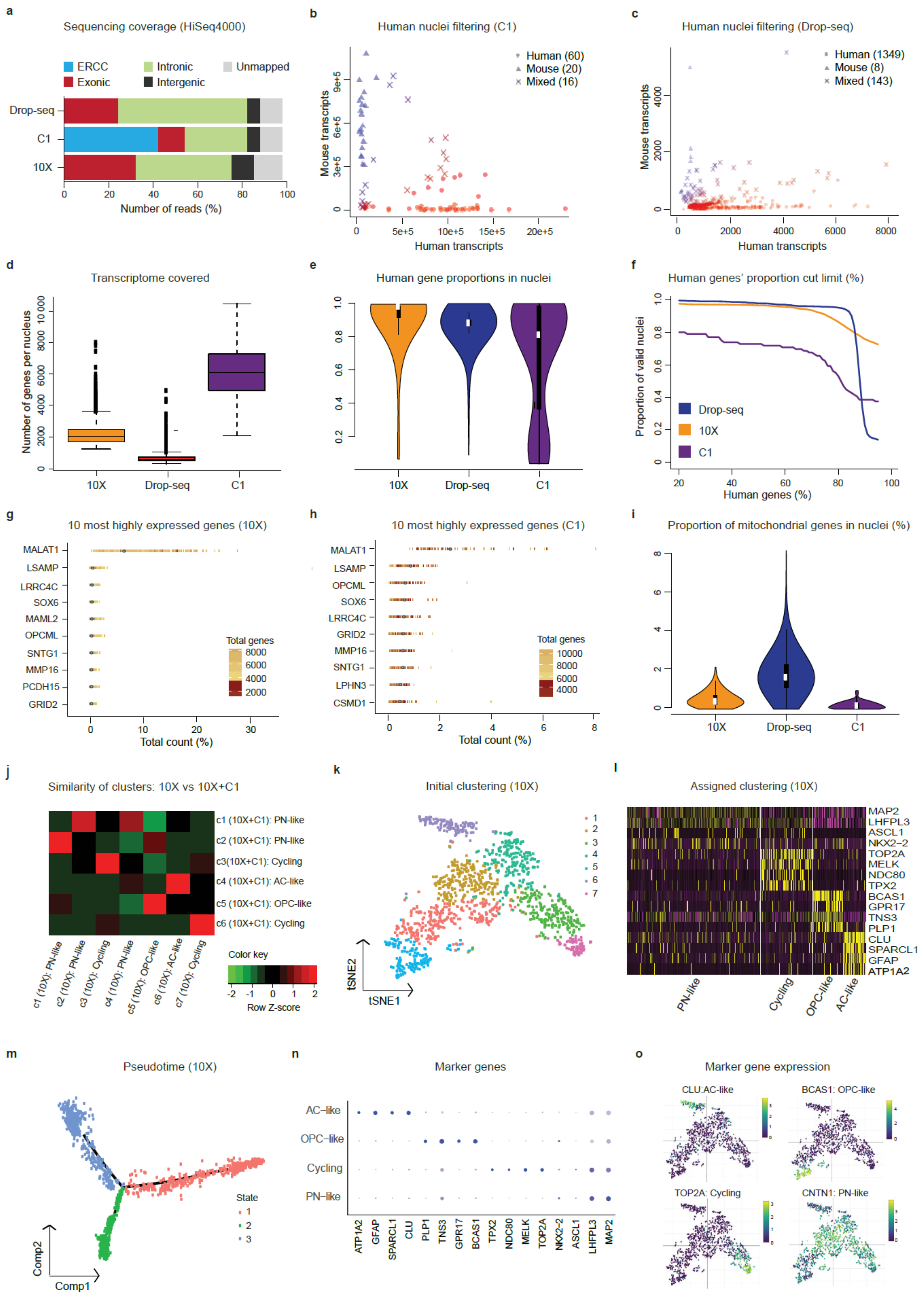
a
Sequencing coverage (HiSeq4000)
ERCC
Exonic
Intronic
Intergenic
Unmapped
Drop-seq
C1
10X
Number of reads (%)
b
Human nuclei filtering (C1)
Human (60)
Mouse (20)
Mixed (16)
Mouse transcripts
Human transcripts
c
Human nuclei filtering (Drop-seq)
Human (1349)
Mouse (8)
Mixed (143)
Mouse transcripts
Human transcripts
d
Transcriptome covered
Number of genes per nucleus
10X
Drop-seq
C1
e
Human gene proportions in nuclei
10X
Drop-seq
C1
f
Human genes' proportion cut limit (%)
Proportion of valid nuclei
Drop-seq
10X
C1
Human genes (%)
g
10 most highly expressed genes (10X)
MALAT1
LSAMP
LRRC4C
SOX6
MAML2
OPCML
SNTG1
MMP16
PCDH15
GRID2
Total genes
Total count (%)
h
10 most highly expressed genes (C1)
MALAT1
LSAMP
OPCML
SOX6
LRRC4C
GRID2
MMP16
SNTG1
LPHN3
CSMD1
Total genes
Total count (%)
i
Proportion of mitochondrial genes in nuclei (%)
10X
Drop-seq
C1
j
Similarity of clusters: 10X vs 10X+C1
c1 (10X+C1): PN-like
c2 (10X+C1): PN-like
c3(10X+C1): Cycling
c4 (10X+C1): AC-like
c5 (10X+C1): OPC-like
c6 (10X+C1): Cycling
c1 (10X): PN-like
c2 (10X): PN-like
c3 (10X): Cycling
c4 (10X): PN-like
c5 (10X): OPC-like
c6 (10X): AC-like
c7 (10X): Cycling
Color key
Row Z-score
k
Initial clustering (10X)
tSNE2
tSNE1
l
Assigned clustering (10X)
MAP2
LHFPL3
ASCL1
NKX2−2
TOP2A
MELK
NDC80
TPX2
BCAS1
GPR17
TNS3
PLP1
CLU
SPARCL1
GFAP
ATP1A2
PN-like
Cycling
OPC-like
AC-like
m
Pseudotime (10X)
State
Comp2
Comp1
n
Marker genes
AC−like
OPC−like
Cycling
PN−like
ATP1A2
GFAP
SPARCL1
CLU
PLP1
TNS3
GPR17
BCAS1
TPX2
NDC80
MELK
TOP2A
NKX2−2
ASCL1
LHFPL3
MAP2
o
Marker gene expression
CLU:AC-like
BCAS1: OPC-like
TOP2A: Cycling
CNTN1: PN-like

Figure S2. Comparison of 10X Genomics, Fluidigm C1 and Drop-seq using a patient-derived xenograft sample shows advantage for 10X Genomics due to high number of nuclei detected. Related to Fig. 2. (a) Proportions of covered reference types from reads alignment of three different snRNA-seq platforms are fairly similar, with the exception of ERCC spike-ins with Fluidigm C1 data. The numbers of human and mouse transcripts per nuclei from of a glioma PDX sample for (b) Fluidigm C1 and (c) Drop-seq data. (d) Boxplot represents numbers of total genes per nuclei between 10X, Drop-seq and C1 platforms. (e) The violin plot represents proportions of human materials per nuclei among platforms. Proportions are computed as a ratio between numbers of human only and total transcripts in a nucleus. (f) Effect of the minimum proportion of human genes cut limit to assign nuclei as valid (not mixed or from mice). Most highly expressed genes were similar in (g) 10X and (h) C1. (i) Proportions of mitochondrial genes per nuclei between 10X, Drop-seq and C1 (before filtering of nuclei with high MT content). (j) Correlation-based comparison of detected clusters between 10X and 10X+C1 combined. (k) t-SNE representation of 10X PDX dataset, original clusters are marked in color. (l) Heatmap of four main assigned cell type specific differentially expressed genes. (m) Original pseudotime trajectory plot before assigning the populations based on (n) high expression of known marker genes (Fig. 2h). (o) Visualization of the assigned cell type marker gene expression in t-SNE representation of 10X PDX.

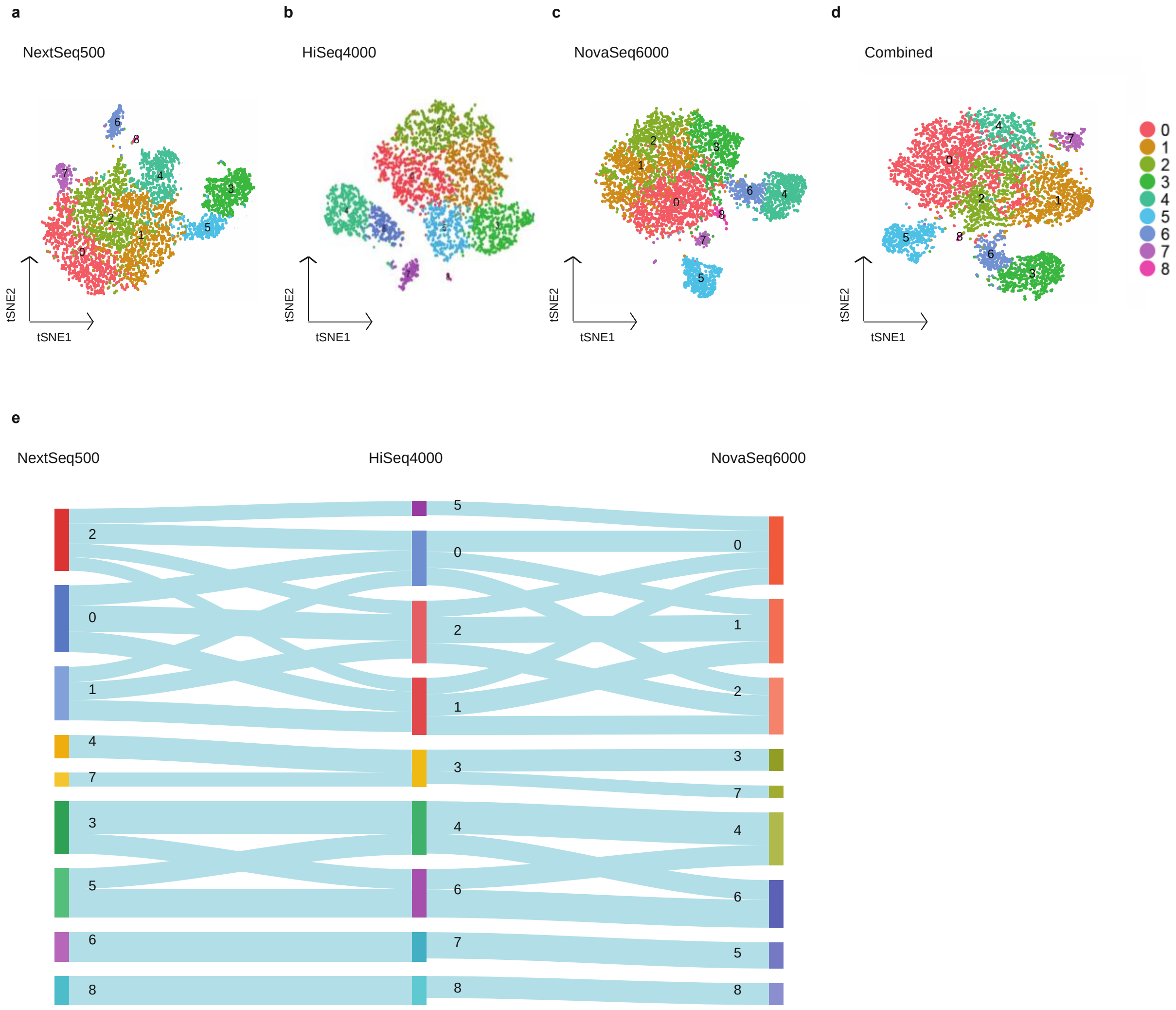

Figure S3. HiSeq4000, NextSeq500 and NovaSeq6000 provide corresponding cluster detection. Related to Fig. 3. T-SNE representations of ICGC_PA56 10X snRNA-seq data sequenced with (a) NextSeq500, (b) HiSeq4000 and (c) NovaSeq6000 resulting in similar cluster detection. (d) Associations between the cell clusters of platforms based on positive correlation limit 0.3 (e) T-SNE representation of ICGC_PA56 snRNA-seq dataset combined from all platforms.

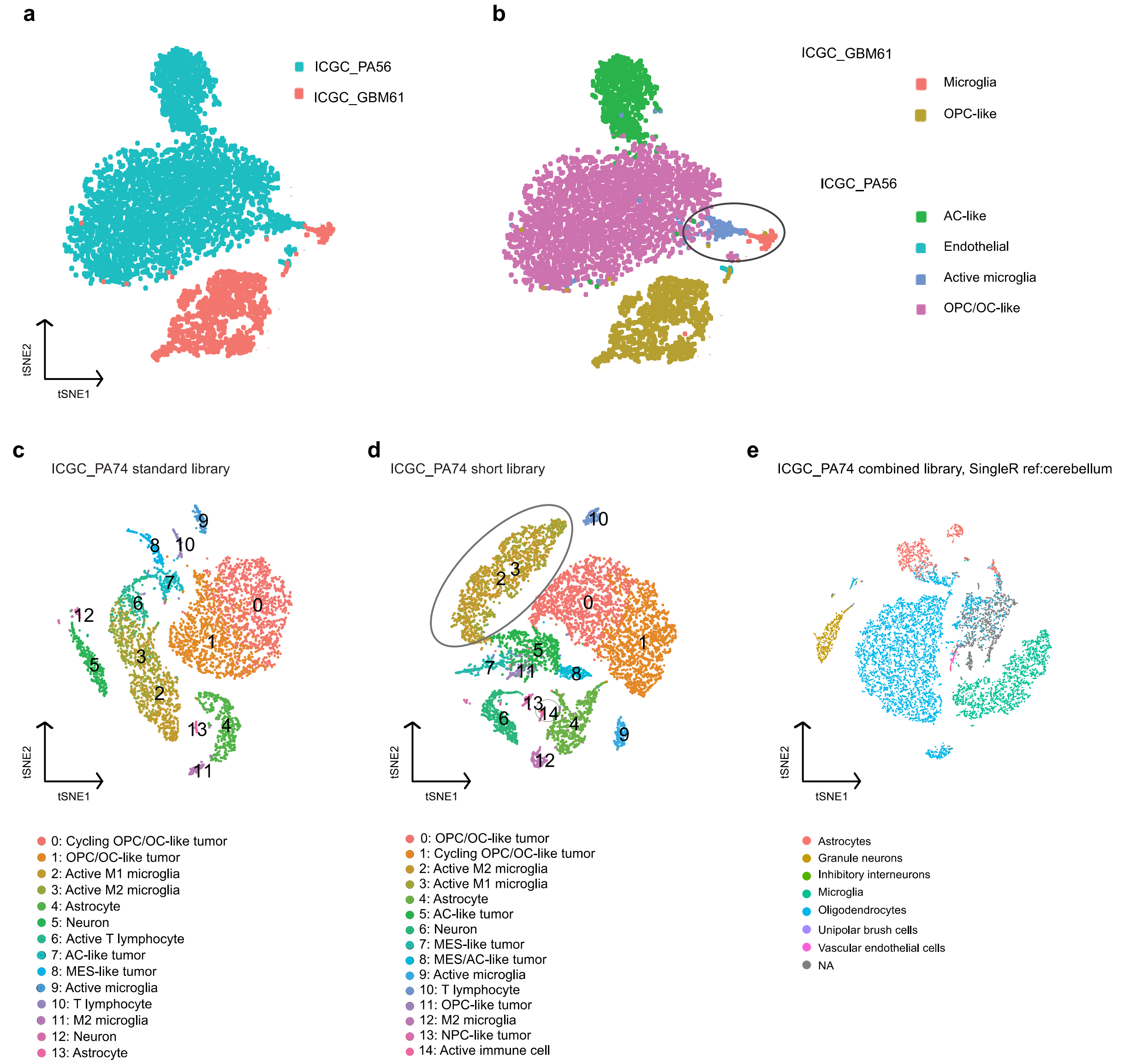

Figure S4. Healthy cell populations from different snRNA-seq tumors cluster proximal to each other and can be detected similarly with both standard and short 10X library size. Related to Fig. 3. (a) ICGC_PA56 and ICGC_GBM61 10X v2 snRNA-seq populations cluster separately as observed in a t-SNE plot. (b) Healthy normal microglia cells (circled) from both tumors cluster close to each other. (c) ICGC_PA74 10X V3.1 snRNA-seq data with standard fragment size of cDNA library and (d) short cDNA fragments in the library. Marker genes unique to the short library were found especially for immune cells (circled). (e) Cell type assignment of SingleR with human healthy cerebellum reference.

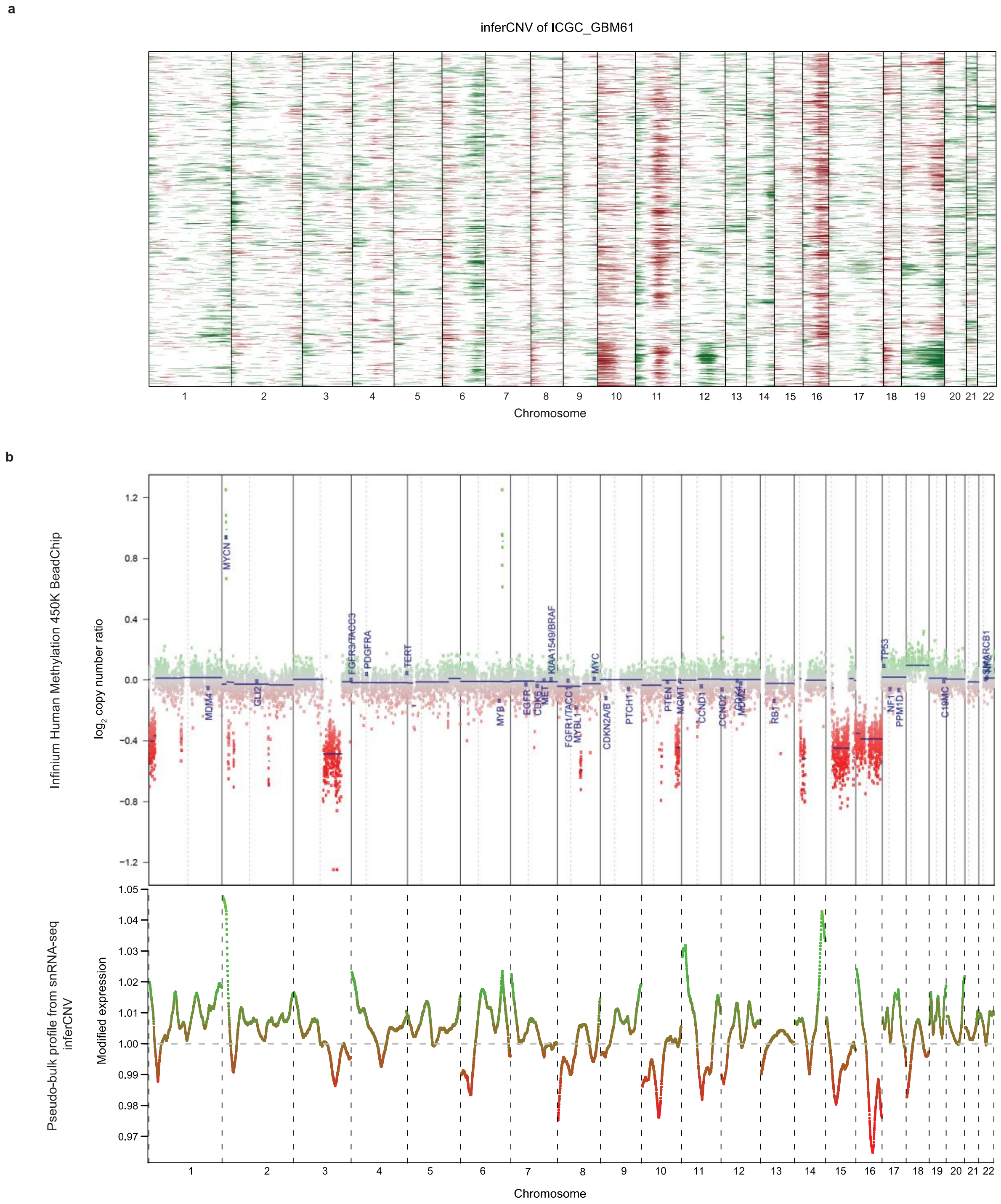

Figure S5. Tumor cells detected from 10X snRNA-seq data using inferCNV analysis. Related to Fig. 4. (a) Copy number variations (CNVs) of single nuclei from 10X snRNA-seq data of ICGC_GBM61 analyzed by inferCNV. (b) A bulk CNV profile of the same tumor derived from Infinium HumanMethylation450 array analysis compared to pseudo-bulk extraction of CNV profiles from 10X data of ICGC_GBM61 using mean values across the cells.

Supplementary tables

Table S1. A rough comparison of the original nucleus isolation method developed by Spalding et. al. (Cell, 2005) and modified by Ernst et. al. (Cell, 2014) and the protocol optimized in this manuscript.

**Nucleus isolation method**	Processing time	Special equipment needed	Cell debris	Nucleus yield
**Sucrose cushion protocol**	2 h	Ultracentrifuge	Substantial	Medium
**Optimized protocol**	<30 min	-	Very low	High

Table S2. Clinical Characteristics and Sample Processing Information of the Pediatric Glioma Tissues Studied by SnRNA-Seq. Related to Figures 2 and 3.

**Patient ID**	B062_004_H1013	ICGC_PA56	ICGC_GBM61	ICGC_PA74	I007_024
**Sample type**	Orthotopic PDX in NSG mouse, in vivo P3	Biopsy	Biopsy	Biopsy	Biopsy
**Age at diagnosis (years)**	15	9	7	7	14
**Gender**	M	M	F	M	F
**Tumor location**	Cerebellum	Cerebellum	Hemispheric	Cerebellum	Frontal lobe
**Diagnosis**	GBM	PA	GBM	PA	PXA
**Genetic alterations**	CDK4 amplification, PDGFRA amplification, TP53 mutation/loss, PTEN loss	KIAA1549:BRAF fusion	MYCN amplification, PLAGL1 amplification	KIAA 1549:BRAF fusion	BRAF V600E, CDK2NA/B loss
**Material**	Frozen cell pellet	Frozen tissue	Frozen tissue	Frozen tissue	Frozen tissue
**Sample frozen (years)**	2	6	5	9	4
**Application**	10X Genomics, v2.0, Fluidigm C1, DropSeq	10X Genomics, v2.0	10X Genomics, v2.0	10X Genomics, v3.1	10X Genomics, v3.1

Table S3. Quality Control Information of the Pediatric Glioma Tissues and the PDX Model Studied by SnRNA-Seq. Related to Figures 2 and 3.

Sample ID	SnRNA-Seq platform	Sequencer (Illumina)	Estimated # of nuclei	Mean reads per nuclei	Median genes per nuclei	Reads mapped to genome (%)	Reads mapped to transcriptome (%)	MT prop. median (%)
ICGC_GBM61	10X v2	HiSeq4000	1458	215117	818	62.1	44.6	0.07
ICGC_GBM61	10X v2	NextSeq500	1546	62305	419	88.7	60.6	0.09
ICGC_GBM61	10X v2	NovaSeq6000	1490	176289	485	76.9	53.2	0.07
ICGC_PA56	10X v2	HiSeq4000	6250	49187	985	88.8	60.1	0.34
ICGC_PA56	10X v2	NextSeq500	6209	11184	669	91	60.6	0.33
ICGC_PA56	10X v2	NovaSeq6000	6283	130198	1164	90.1	60	0.34
B062_004_A1013	10X v2	HiSeq4000	2666	121837	2172	86.1	75.5	0.57
B062_004_A1013	Fluidigm C1	HiSeq4000	78	2909145	6107	47.8	41.6	0.32
B062_004_A1013	DropSeq	HiSeq4000	1353	11027	663	89.5	83.3	1.24
ICGC_PA74 standard	10X v3.1	NovaSeq6000	11123	76457	3666	95.8	56.6	0.54
ICGC_PA74 short	10X v3.1	NovaSeq6000	12565	108547	3301	84.5	47.3	0.36
ICGC_PA74 combined	10X v3.1	NovaSeq6000	13318	166266	4293	91.2	51.5	0.55
I007_024 standard	10X v3.1	NovaSeq6000	13906	48159	3439	96.4	51.6	0.30
I007_024 short	10X v3.1	NovaSeq6000	13702	92737	3758	88.8	41	0.13
I007_024 combined	10X v3.1	NovaSeq6000	17098	113486	4509	94.6	45.5	0.26

Table S6. The optimal volume of nuclei storage buffer to use for the final re-suspension of the isolated nuclei.

Pellet size of isolated nuclei	Final re-suspension volume (ul)
Barely visible	50-70
Thin smear on the tube wall	70-100
Clear smear on the tube wall	100-200
<1 mm thick pellet	200-400
1-2 mm thick pellet	400-700
2-3 mm thick pellet	700-900
>3 mm thick pellet	1000

As separate Excel files:

Table S4. Upregulated genes per each cluster. Related to Figures 2, 3 and 4.

Table S5. Enrichment analysis of specific upregulated DEGs based on GSEA Msigdb 7.0 database using hypergeometric test. Related to Figures 2 and 3.
